# Evaluating the COVID-19 impact in Italian regions via multi criteria analysis

**DOI:** 10.1371/journal.pone.0285452

**Published:** 2023-05-10

**Authors:** Francesca Santucci, Martina Nobili, Luca Faramondi, Gabriele Oliva, Bianca Mazzà, Antonio Scala, Massimo Ciccozzi, Roberto Setola

**Affiliations:** 1 Unit of Automatic Control, Department of Engineering, Università Campus Bio-Medico di Roma, Rome, Italy; 2 ISC-CNR Physics Department, Università La Sapienza, Roma, Italy; 3 Global Health Security Agenda – GHSA Italy; 4 Medical Statistic and Molecular Epidemiology Unit, University of Biomedical Campus, Rome, Italy; Libyan Academy, LIBYA

## Abstract

Italy was the first European country to be significantly impacted by the COVID-19 pandemic. The lack of similar previous experiences and the initial uncertainty regarding the new virus resulted in an unpredictable health crisis with 243,506 total confirmed cases and 34,997 deaths between February and July 2020. Despite the panorama of precariousness and the impelling calamity, the country successfully managed many aspects of the early stages of the health and socio-economic crisis. Nevertheless, many disparities can be identified at the regional level. The study aims to determine which aspects of regional management were considered more important by the citizens regarding economic and health criteria. A survey was designed to gather responses from the population on the Italian regions’ response and provide a ranking of the regions. The 29-item online survey was provided to 352 individuals, and the collected data were analyzed using the Analytic Hierarchy Process methodology. The results show a general agreement in considering of greater relevance the healthcare policies rather than the economic countermeasures adopted by regional governments. Our analysis associated a weight of 64% to the healthcare criteria compared to the economic criteria with a weight of 36%. In addition to the results obtained from the Analytic Hierarchy Process, the sample’s composition was analyzed to provide an overall assessment of the Italian regions. To do so, we collected objective data for each region and multiplied them by the overall weight obtained for each sub-criteria. Looking at the propensity to vaccination or the belief in a relation between COVID-19 and 5G according to age and educational qualification helps understand how public opinion is structured according to cultural and anthropological differences.

## Introduction

In late 2019 a newly discovered virus, belonging to the SARS-CoV strain, caused a cluster of pneumonia cases in Wuhan (China). The virus rapidly spread across countries causing a global pandemic that is still ongoing. Within a couple of months, coronavirus disease (COVID-19) led to 40,598 deaths worldwide, with a case fatality rate (CFR) of 4.9%. [1] In this rapidly changing scenario, various governments started to adopt a series of restrictive measures in conjunction with healthcare interventions to delay and weaken the spread of the virus. Italy was among the first countries to face the COVID-19 outbreak, with the first confirmed case detected in Lombardia on February 21^st^, 2020. Since early March, the Italian government started to implement restrictive measures to limit people’s interactions and, consequently, the virus transmission. Italy was the first country to place its entire national territory under a lockdown, beginning on the 10^th^ of March 2020. Freedom of movement was re-established only on May 4^th^, 2020, and was accompanied by a series of mandatory non-pharmaceutical interventions, such as bans on public events, schools closure, the mandatory wearing of medical masks, active surveillance of clusters, and hygiene recommendations [2–4]. At the same time, new field hospitals were built throughout the whole nation as a response to the rapidly increasing demand for new intensive care units (ICUs) able to host such a high number of ventilator-dependent patients. The country’s steps to isolate affected areas showed how drastic and timely restrictions were necessary to effectively limit the levels of infection and ease the burden of the cases [5].

During this unprecedented emergency situation, Italy’s experience and strict containment measures were taken as a guideline by several European countries [6]. Indeed Italy, while perpetrating some inevitable mistakes, was successful in managing many aspects of the health and socio-economic crisis. On one hand, the government acted promptly to contrast the virus transmission, and Italian scientists succeeded in isolating the DNA sequence of the coronavirus, giving a decisive contribution to deepening the knowledge of the unknown disease and providing new insights into the mutagenic capabilities of the virus [7–9]. On the other hand, the inadequacy of ICUs to accommodate the exceptional number of patients alongside the lack of knowledge on the disease initially led to the loss of many lives [10]. From an economic perspective, the closure of industries and non-essential business activities for a prolonged period caused an extended economic crisis. In addition, the enforcement of restrictive rules in a western democracy led to a general discontent among the population, worsened by the fact that lockdown isolation enhanced the likelihood of developing mental health disorders (e.g., depression) [11].

However, being the Italian health system a federated one where responsibilities are demanded to regions, there were significant differences among the actual and perceived efficacy of the adopted measure from one region to another.

### Contribution

The aim of our study is to assess how the COVID-19 management policies of the various Italian regions, undertaken during the first stages of the pandemics, were welcomed and assessed by the residents, on the basis of health and economic criteria. Indeed, Italian regions have been affected to different extents by the pandemic crisis, thus, within the national guidelines, each region developed its own response plan and mitigation strategy [12]. The evaluation was performed by presenting a questionnaire to 352 individuals coming from different regions. From the collected data we obtained an overall evaluation of the Italian regions with respect to the quality of their responses to the emergency. Such ranking is defined on the basis of a multi-criteria decision-making approach, namely the Analytic Hierarchy Process (AHP) [13]. The AHP approach is one of the most used and accurate multi-criteria decision methods that find application in many fields, such as energy optimization [14], infrastructures protection [15], and plant construction [16]. It is particularly useful when there is a need to evaluate a large number of alternatives according to several criteria [17]. Such methodology, thanks to the hierarchical approach, is able to estimate an absolute ranking for a set of alternatives on the basis of relative comparison provided by experts according to multiple criteria.

A preliminary analysis was conducted to assess the internal (i.e., age, status of employment, cultural background) and external influences of the subjects that completed the questionnaire: to better understand their stance on issues such as the propensity to get vaccinated and susceptibility to fake news spread by the mass media [18].

The aim of the proposed study is to evaluate the COVID-19 pandemic event management of the Italian regions based on the population opinion combined with objective data. Starting from the AHP evaluation of healthcare and economic criteria, we estimate an overall evaluation of its pandemic management for each region.

The paper is structured as follows: in the section *Related Works* we present the main studies on the socio-economic impact of COVID-19 on Italian regions, in the *Proposed Evaluation Scheme* section we define the proposed evaluation methodology based on the AHP. In *Questionnaire Design* we illustrate the various items of the questionnaire that was submitted to individuals and in *Description of the Sample* we provide the characteristics of the statistic sample. In *Results* we illustrate the results obtained from the analysis of the preliminary information about the sample composition, collected through the questionnaire, and the outcome of the multi-criteria analysis on the healthcare and economic aspects obtained using the AHP methodology.

## Related works

Since its outbreak, the ramifications of COVID-19 across the globe have been thoroughly studied at the international level, ranging from its impact on the environment [19], tourism decline [20] and the psycho-social dimension [21–24]. Particular attention has been reserved to the socio-economic consequences that national lockdowns left behind and for the healthcare management issues and flaws on which the COVID-19 emergency shone a light [25–30]. When evaluating COVID-19 impact on healthcare services, it is important to consider its burden on medical personnel, the availability of places in the wards, the number of ICUs, and the provision of vaccine. Therefore, the critical responsibility of the governments was to make swabs available to diagnose the disease and to raise awareness of the importance of vaccination to reduce crowding in hospitals. From the economic point of view, it is important to consider both the short-run costs (e.g., losses of human and physical capital, pauses in processes of consumers and producers) and the long-run costs (e.g., permanent health problems of survivors, closure or distortion of many business activities, a temporary decrease in productivity). According to the study presented in [26], the economic impact of coronavirus has been more severe in Europe and America compared to Asian economies. In this context, the most important duty of governments was to guarantee financial support to the lower and middle classes, more affected by the economic meltdown. Recently, a few studies have raised the issue of providing an evaluation of the country’s governments’ response to the pandemic in terms of both economic and healthcare management [31–33]. As for Italy, a few papers focused on critically reviewing the country’s response to the COVID-19 crisis, debating to which extent the government’s management was responsible for the territorial concentration of the virus and the high mortality rate [34–38].

According to recent work from Aristei et. al [35], Italy responded promptly to the emergency state with the creation of specific task forces of technical experts and scientific support bodies at the national (ISS, AIFA) and regional levels. These commissions provided support and scientific advice to the government in the allocation of significant economic resources (EUR 3.7 billion in 2020 and EUR 1.7 billion in 2021) to enhance epidemiological surveillance, the research activities, to increase hospital facilities and ICU beds, and to create custom ICUs to manage COVID-19 patients establishing an ad-hoc COVID-19 integrated surveillance system. However, several elements, mainly related to the fact that Italy has 20 different regional healthcare systems, hindered the attempt to implement a rapid and coordinated response. More in detail, the weak coordination with regional bodies, led to a frail and uneven response, with some regions implementing autonomous policies (testing, contact tracing, containment measures) not always in line with the central government. In some cases, the lack of coordination between hospital and primary care and territorial services resulted in an inefficient response (e.g., saturation of hospitals and inability to manage patients), and the lack of previously structured digital health tools reduced the possibilities of safe remote management of mild symptomatic cases. Furthermore, Italy showed several limits in the capacity planning of hospitals, which highlighted the need to develop models for hospital surge capacity planning.

However, Italy demonstrated good long-term organizational skills in regard to the design of a four-risk scenarios plan developed by the task force of experts in collaboration with the Italian technical-scientific bodies at the end of the first wave of the pandemic. This plan considered specific indicators of probability (e.g., virus transmission capacity, spread in working environments), impact on hospitals (occupancy of beds in ICUs), and resilience (e.g., degree of acceptance of hygiene tips and face masks) to subdivide and classify regions in 4 areas on the basis of the risk level (i.e., white, yellow, orange, and red). In this way, after the first national lockdown, varying degrees of restrictive measures were strategically applied to high-risk areas, limiting the negative economic consequences of stricter restrictions to only certain regions. However, Aristei et al. highlighted how the constant lack of adequate funding for the National Health Service in Italy over the years has led not only to the lack of sufficient healthcare workers, structures, and technologies but also to the absence of an integrated management system and accepted rules [35].

Contributing to the debate, Ricci et al. focused on evaluating medical responsibility and the judgment of government measures [36]. The authors argued that any government policy assessments should not be limited to considering part of their liability the occurrence cases of willful misconduct, since during an emergency the possibility of errors is exponentially heightened. In an outbreak context, there are no established guidelines or good practices, and there are no foreseeable aids to measure diligence and responsibility. Thus, when assessing the effective government measure, the evaluation should disproportionately be influenced by the analysis of cases of gross negligence by doctors, willful misconduct, or fault for employer liability. Indeed, in a scoreboard by the Deep Knowledge Group study which analyzes the capacity, scope, diversity, efficiency, and effectiveness of government measures to provide economic support to citizens and businesses, Italy ranked in the tenth place in the world for its resilience [39].

While the previous statements are the result of the critical analysis of publicly available objective data on the socio-economic situation in Italy, it is also critical to address the citizen’s perspective. The work of Felice et al. [37], centered around the opinion of the population through a 40-item e-survey named “Impact of COVID-19 outbreak on healthcare workers in Italy”, designed through an online platform (“Online surveys”, developed by the University of Bristol [40]) and made available from March 25th to April 4th, 2020. The survey, despite the sensitivity to regional differences, aimed at assessing only crucial elements in the experience of healthcare workers. Thus, it did not provide insights into the economic aspects of the emergency and interviewed only a relatively small category of the population. The survey demonstrated profound variations across high- and low-prevalence regions, specialty sectors, and professional figures.

In this study, we decided to evaluate Italian regions’ response to the first stages of the pandemic emergency with respect to popular opinion, thus we designed and made available a 29-item online survey that allowed to collect the responses of 352 individuals chosen randomly from all the 20 different regions (following the sole criterion of having more people where the population density is higher). The data was analysed using a multi-criteria decision-making methodology to evaluate the popular opinion with respect to both the economic and healthcare-related aspects.

Several potential approaches are applicable when it is required to evaluate multiple alternatives considering multiple criteria. In this work, among others, the Analytic Hierarchy Process seems to hold some characteristics requested to facilitate the data collection. The Analytic Hierarchy Process (AHP) is one of the most used and accurate Multi-Criteria Decision Method (MCDM). It is frequently used for its flexibility in combining relative judgments with the aim of providing an absolute evaluation of several alternatives according to multiple criteria. AHP allows to reduce the number of relative comparisons thanks to the adoption of a hierarchical structure and, in addition, it allows to manage also incomplete information in its sparse setting. Several approaches were developed to solve similar problems. The Interpretive Structural Modelling (ISM) [41] is an interactive approach that allows to identify the relationship between specific items that define the problem. This method is widely used in the scheduling of activities where a systematic prioritization of the characteristic of a problem is fundamental. In this approach, the subjects should express an opinion on each alternative and this aspect increases the time required to obtain the results and, at the same time, stresses the decision-makers. The DEcision MAking Trial and Evaluation Laboratory (DEMATEL) [42] method is a valid approach for the identification of cause-effect chain components of a complex system. Such a method aids in creating connections between obstacles or issues based on expert evaluations. The TOPSIS approach [43] is a method of compensatory aggregation that compares a set of alternatives in order to identify the best alternative. It is based on the concept that the chosen best alternative should have the shortest geometric distance from the positive ideal solution and the longest geometric distance from the negative ideal solution. It permits obtaining the best criterion to optimize the problem and reduce the negative aspect. The problem related to this method is the identification of the positive and negative ideal solution, indeed it is usually used in combination with other methods like AHP. An AHP-similar method is the Best-worst multi-criteria decision-making method [44]. Some of these methods, as well as AHP, are applied to analyze the COVID-19 pandemic event [45] but the features of the AHP in terms of simplicity and ease of use make it the best choice for this study. More precisely, the adoption of a hierarchy structure, the possibility to consider also incomplete data (i.e., when a decision maker does not provide some relative judgments), and the presence in the literature of multiple metrics for the consistency check, make the AHP the best approach for our study. According to the comparative analysis proposed in [46], which considers the AHP, ANP; TOPSIS, and DEMATEL approaches, the AHP and the ANP are the only methods based on pairwise comparison and at the same time include a validation step based on the consistency of the given relative comparisons.

In the literature, a lot of studies adopt MCDM approaches in order to evaluate some phenomena related to the COVID-19 pandemic. Shadeed et al. [47] presented a map of the vulnerabilities correlated with the COVID-19 pandemic event for the West Bank region in Palestine. The study aims to propose a map to guide and contribute to the prediction of potential explosions of the virus. In this way, the authorities have the possibility to prevent and mitigate the consequence on the public healthcare system. The map was realized based on the use of AHP weight obtained comparing parameters like the population in the area, the density of population, food and accommodation services, chronicle illness, etc. The obtained risk map was compared with objective data produced and spread by the region. In [48], Requia et al. proposed a study to evaluate the healthcare system in over 5572 municipalities in Brazil due to COVID-19. The study was realized combining the AHP method with the GIS technique to analyze the different geographic regions. The model was compared with the objective data like injection rate and hospitalization rate. Das et al. in [49] propose a study based on the AHP with the aim to analyze the vulnerabilities of the slums area of India considering the pandemic event one of the main critical aspects. The slum areas are vulnerable areas for different reasons: floods, malaria, blazes, and now pandemic event like COVID-19. They analyzed with the AHP method 4 different macro-criteria: percentage of slums, social distance, family status, and slums mobility. For each macro-criteria, they considered 3 sub-criteria, for a total of twelve elements. Another study that involved the Indian situation related to the COVID-19 pandemic event was proposed by Choudhury et al. [50]. The aim of the study was to evaluate the Indian states’ preparation for the pandemic event. They considered different types of parameters: demographic, socioeconomic, and healthcare parameters. For each of these macro-criteria, they considered 3, 3, and 4 sub-criteria, for a total of 10 parameters evaluated. They used the fuzzy AHP approach to evaluate the criteria weight to analyze the performance. Their idea was to consider the different counties of India like a cluster, in this way, the government could evaluate a specific strategy to mitigate the effect of COVID-19. At the end of the study it came to light that healthcare parameters were considered more relevant than the others. Moreover, it emerged that the South countries had a better preparation to contrast and mitigate the pandemic than the other regions. An additional study that used the AHP approach to evaluate the COVID-19 event was proposed by Gao et al. in [51], where they proposed an AHP regional model to analyze the vulnerabilities of the 4 principal cities in China. They collected data and combined it with objective data related to the injection from the cities to obtain a regional vulnerabilities model. The authors evaluated the AHP weight for 3 main criteria: pathological, medical, and response attributes. Based on the calculated data and the objective ones they realized a scalable model applicable both for the cities analyzed and for different regions and cities of the country.

## Proposed evaluation scheme

**Fig 1:**
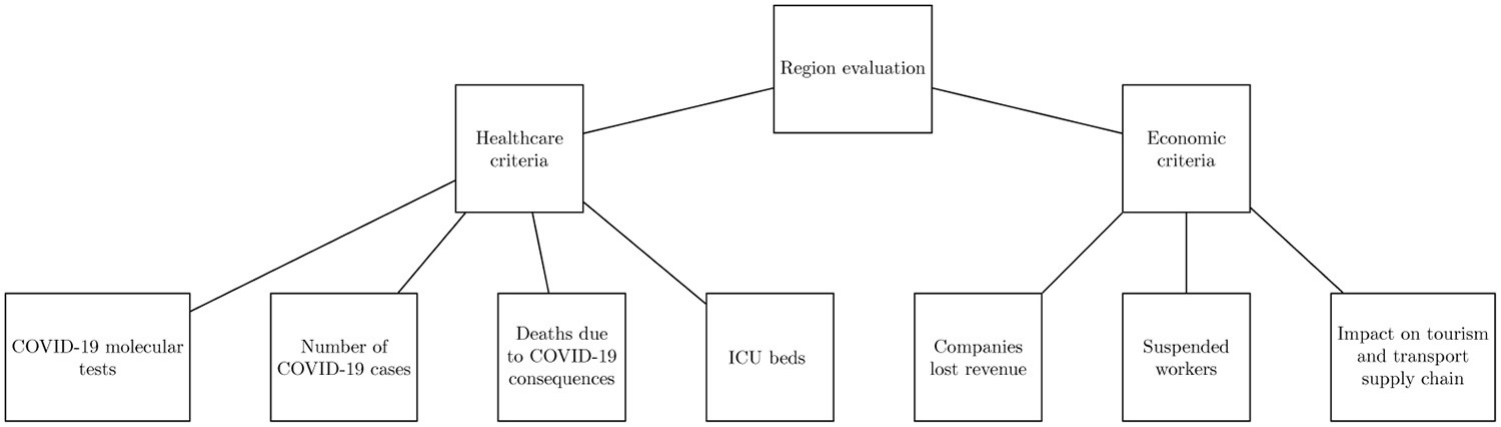
Decomposition of the problem into a hierarchy.

As mentioned above, the aim of this work is the evaluation of the Italian regions’ response to the pandemic emergency with respect to multiple criteria and sub-criteria by considering the perspective of the population. As depicted in the hierarchical structure in Fig 1, we evaluate the efficiency of the Italian regions counteracting the spread of COVID-19 by considering two criteria: the *economic perspective* and the *healthcare-related aspects*. In this way, we want to include in our evaluation the direct effects of the diseases related to the pandemic circumstances and the economic effects of regional restrictive policies aimed to limit the outbreak. Moreover, for each criterion, we consider a set of sub-criteria. More precisely, considering the healthcare perspective, in our analysis we consider the following sub-criteria. The *number of COVID-19 cases*, the number of *molecular tests* (nasal and oropharyngeal swabs), the number of *deaths* due to COVID-19 consequences, and the number of available *intensive care unit (ICU) beds*. On the other side, concerning the evaluation criteria related to the economic perspective, we take into account the sub-criteria related to the *number of suspended workers* due to the national and regional lockdown, the *impact on tourism and transport supply chain*, and the companies *lost revenue* (both expressed in terms of percentage loss against 2019). In such a multi-criteria context, our aim is to define a holistic evaluation of the Italian regions’ ability to cope with the COVID-19 epidemic. With the aim to consider all these heterogeneous perspectives in the evaluation process, we now formalize our approach based on the AHP and the *Incomplete Logarithmic Least-Squares* (ILLS) problem (for further details on the method see *Appendix 1—Preliminaries*) in order to compute the global weights **w**_1_,…, **w**_7_ associated to each sub-criteria (see Table 1). Such a preliminary step about the definition of a weight for each sub-criteria, is essential in order to provide an overall evaluation for each region considering multiple heterogeneous aspects. As mentioned before, we iteratively apply the ILLS approach to each level of the hierarchical structure depicted in Fig 1 in order to estimate the weights of each criterion and sub-criteria. Starting from the highest level of the hierarchical structure, the preliminary step is the identification of the local weight of each set of criteria and sub-criteria. Let $M^{\left(u\right)}\in R^{2\times 2}$ be the comparison matrix provided by the *u*-th questionnaire respondent with respect to the two criteria at the highest level of the hierarchy (i.e., healthcare and economic aspects), and let $M_{ij}^{\left(u\right)}$ be the relative relevance of the criteria *i* with respect to the criteria *j* for the *u*-th questionnaire respondent, defined according to the Saaty’s scale (see [13] for additional details). Similarly, with the aim to compute the local weights associated with each sub-criteria, let $H^{\left(u\right)}\in R^{4\times 4}$ and $E^{\left(u\right)}\in R^{3\times 3}$ be respectively the comparison matrices provided by the *u*-th questionnaire respondent collecting the relative scores about the healthcare and economic sub-criteria respectively. The preliminary step is the identification of the local weights $m\in R^{2}$, $h\in R^{4}$, and $e\in R^{3}$ respectively derived by the matrices M,H, and E on the basis of Eq (3). Note that the local weights of criteria and sub-criteria are normalized between 0 and 1 with a sum equal to 1. For the sake of clarity, in Table 1, we summarize the list of criteria and sub-criteria and the associated notation.

**Table 1:** List of considered sub-criteria.

Criteria	Sub-criteria description	Criteria Local Weights	Sub-Criteria Local Weights	Global Weighs
Healthcare	Number of COVID-19 molecular tests	**m**_1_	**h**_1_	**w**_1_ = **m**_1_**h**_1_
Healthcare	Number of COVID-19 cases	**m**_1_	**h**_2_	**w**_2_ = **m**_1_**h**_2_
Healthcare	Number of deaths due to COVID-19 consequences	**m**_1_	**h**_3_	**w**_3_ = **m**_1_**h**_3_
Healthcare	Number of ICU beds	**m**_1_	**h**_4_	**w**_4_ = **m**_1_**h**_4_
Economic	Companies lost revenue	**m**_2_	**e**_1_	**w**_5_ = **m**_2_**e**_1_
Economic	Number of suspended workers	**m**_2_	**e**_2_	**w**_6_ = **m**_2_**e**_2_
Economic	Impact on tourism and transport supply chain	**m**_2_	**e**_3_	**w**_7_ = **m**_2_**e**_3_

Due to the hierarchical structure of the proposed approach, according to the procedure described in [52], it is possible to compute the global weights as summarized in the last column of Table 1. Such global weights, associated with each sub-criteria, are computed on the basis of the local weights (i.e., the solution of ILLS) and correspond to the product of the local weight of each sub-criteria and the local weight of the associated criteria.

For each Italian region *i* (which represents an alternative) we consider the vector $r^{\left(i\right)}\in R^{n}$, which collects the real numerical values considering the *i*-th Italian region for each considered sub-criteria (for instance, $r_{j}^{\left(i\right)}$ represents the real numerical value of the *i*-th alternative (region) according to the *j*-th sub-criteria). For example, $r_{1}^{\left(i\right)}$ and $r_{2}^{\left(i\right)}$ respectively represent the number of COVID-19 cases and the number of molecular tests associated with the *i*-th region.

Once the global weights are computed, the overall evaluation of the *i*-th region *g*^(*i*)^ is finally computed as a weighted index:

$$\begin{array}{c} \begin{array}{cc} g^{\left(i\right)}= & \;m_{1}h_{1}r_{1}^{\left(i\right)}+m_{1}h_{2}r_{2}^{\left(i\right)}+m_{1}h_{3}r_{3}^{\left(i\right)}+m_{1}h_{4}r_{4}^{\left(i\right)}+m_{2}e_{1}r_{5}^{\left(i\right)}+m_{2}e_{2}r_{6}^{\left(i\right)}+m_{2}e_{3}r_{7}^{\left(i\right)}\\ = & \;r^{\left(i\right)}w. \end{array} \end{array}$$ (1)

### Dataset description

As mentioned above, the aim of this work is to evaluate the Italian regional response to the pandemic emergency, considering both healthcare and economic criteria. The relevance of each sub-criterion in the final evaluation is computed according to the procedure described in the previous section. In this section, we summarize the main aspects related to the data used in the evaluation process. More precisely, we now discuss some relevant remarks regarding the characteristics of such data, the selected sources, and the procedure required for the normalization. According to the previously introduced notation, we remark that $r_{j}^{\left(i\right)}$ represents the numerical value of *i*-th region on the basis of sub-criterion *j*. All data involved in the evaluation process are collected in Table 2. Notice that, the numerical values in Table 2 represent the evaluations of the *i*-th region with respect to each sub-criteria, such evaluation will be considered according to Eq (1) in order to provide the overall evaluations *g*^(*i*)^. Such numerical data provide a snapshot of the first stages of the COVID-19 pandemic, it takes into consideration the health data (e.g., cases, tests, etc. provided by the Ministry of Health [54] and the National Agency for Regional Health Services [53]) over a period from the beginning of the pandemic until the end of June. As for the economic data, these are projections and estimates produced by ISTAT [56] e Cerved [55]. Regarding the healthcare data characterizing the context of the pandemic, for each region, Table 2 collects the number of total cases, the number of tests performed (considering the sum of rapid and molecular tests), the number of deaths caused by COVID-19, and the number of planned ICU places. Note that in order to obtain ratings in the range [0, …, 1], each entry in the table is normalized in that range. Also note that an evaluation of 1 is always associated to a positive evaluation, whereas an evaluation of 0 is understood to be negative. For this purpose, normalization was performed considering these factors and the number of inhabitants in the specific region. In the table, the normalized values of each sub-criterion are shown in parentheses. A preliminary analysis of the health data shows that the highest mortality rate relative to the regional population was obtained in Lombardia and Valle d’Aosta. These are respectively the least and most populated regions in Italy. The number of deaths (compared to the regional population) is very high in Lombardia with 16640 representing 47.89% of total deaths in Italy. As for the number of intensive care beds (compared to the regional population), there was a high availability of places in Emilia Romagna, Valle d’Aosta, and Toscana. With regards to virus-tracking activities (number of tests performed compared to population density), Veneto is the region that performed the highest number of tests for virus detection, while the southern Italian regions (Sardegna, Calabria, Campania, Puglia, and Sicilia) performed a low number of tests with respect to their large population sizes, also because the first COVID-19 cases were concentrated in the North of the Country. This aspect surely had an impact on the global evaluation of the regions’ COVID-19 management by the interviewed sample in this study. In terms of economic parameters, the number of suspended workers is expressed as a percentage of the total number of workers employed in the specific region. The result is that the most affected region, according to the forecast made by ISTAT [56], was Marche, followed by Piemonte, Veneto, Basilicata, Sardegna, and Abruzzo. The first two regions are strongly linked to the automotive market, which was suspended and thus was not included in the list of essential services delineated by the laws of March 11 and 22, 2020. The impact of COVID-19 on the tourism and transportation supply chain (that includes several sectors such as airplane transport, local public, maritime, rentals, travel, hotels, and catering) is expressed as a percentage reduction in the turnover of enterprises operating in the tourism and transportation supply chain compared to the previous year. It estimates a loss of 22 billion in 2020 and 7 billion in 2021 compared to pre-Covid forecasts. Sardegna, which bases its economy on tourism, resulted to be the most affected. Finally, the turnover lost by regional companies was considered, again expressed as a percentage loss in comparison to the pre-Covid projection. In this scenario, Piemonte reported the most significant losses due to the presence of many companies in the automotive sector.

**Table 2:** Numerical dataset.

*i*	Region	Tests		Cases		Deaths		ICU beds		Lost Revenue		Suspended Workers		Impact on Tourism	
		[53]	$r_{1}^{\left(i\right)}$	[53]	$r_{2}^{\left(i\right)}$	[53]	$r_{3}^{\left(i\right)}$	[54]	$r_{4}^{\left(i\right)}$	[55]	$r_{5}^{\left(i\right)}$	[56]	$r_{6}^{\left(i\right)}$	[57]	$r_{7}^{\left(i\right)}$
1	Abruzzo	69874	(0.4132)	3292	(0.7358)	462	(0.787)	151	(0.5337)	-9.9	(0.1081)	34	(0.1905)	-20.6	(0.1922)
2	Basilicata	39294	(0.3609)	402	(0.9248)	27	(0.971)	64	(0.5271)	-11.1	(0)	30	(0.2857)	-21.6	(0.1529)
3	Calabria	94170	(0.25)	1180	(0.9362)	97	(0.9699)	221	(0.5261)	-7	(0.3694)	26	(0.381)	-19.8	(0.2235)
4	Campania	279246	(0.2489)	4666	(0.9154)	431	(0.9551)	600	(0.4794)	-7	(0.3694)	32	(0.2381)	-23.7	(0.0706)
5	Emilia-Romagna	489451	(0.5675)	28472	(0.328)	4255	(0.4231)	962	(1)	-6.7	(0.3964)	36	(0.1429)	-19.7	(0.2275)
6	Friuli Venezia Giulia	187500	(0.7977)	3308	(0.7135)	345	(0.8284)	155	(0.5913)	-6.6	(0.4054)	38	(0.0952)	-17.7	(0.3059)
7	Lazio	335751	(0.2953)	8105	(0.8549)	837	(0.9139)	707	(0.5575)	-8.5	(0.2342)	26	(0.381)	-17.1	(0.3294)
8	Liguria	145902	(0.4865)	9974	(0.323)	1558	(0.3925)	251	(0.7504)	-7.7	(0.3063)	30	(0.2857)	-18.6	(0.2706)
9	Lombardia	1030431	(0.5295)	93839	(0.0183)	16640	(0)	1260	(0.5806)	-6.6	(0.4054)	38	(0.0952)	-21.9	(0.1412)
10	Marche	136385	(0.4623)	6785	(0.5318)	991	(0.6072)	163	(0.4954)	-7.6	(0.3153)	42	(0)	-23.8	(0.0667)
11	Molise	22474	(0.3802)	445	(0.8468)	23	(0.9545)	27	(0.4095)	-7.3	(0.3423)	32	(0.2381)	-21.1	(0.1725)
12	Piemonte	411939	(0.4889)	31338	(0.2429)	4087	(0.4328)	420	(0.4469)	-9.8	(0.1171)	38	(0.0952)	-20.4	(0.2)
13	Puglia	175811	(0.2256)	4531	(0.8816)	543	(0.9185)	306	(0.3521)	-6.9	(0.3784)	31	(0.2619)	-20.3	(0.2039)
14	Sardegna	82478	(0.2601)	1364	(0.9124)	132	(0.9513)	163	(0.4609)	-8.9	(0.1982)	28	(0.3333)	-25.5	(0)
15	Sicilia	206550	(0.2136)	3078	(0.9352)	281	(0.966)	611	(0.5665)	-7.8	(0.2973)	24	(0.4286)	-20.2	(0.2078)
16	Toscana	334198	(0.4633)	10248	(0.7108)	1104	(0.821)	650	(0.8079)	-7.2	(0.3514)	37	(0.119)	-21.9	(0.1412)
17	Trentino A.A.	203897	(0.9831)	7502	(0.2637)	697	(0.607)	124	(0.5361)	-8.1	(0.2703)	30	(0.2857)	-24.3	(0.0471)
18	Umbria	94519	(0.5541)	1440	(0.8282)	80	(0.9452)	96	(0.5045)	-5.4	(0.5135)	33	(0.2143)	-21.3	(0.1647)
19	Valle d’Aosta	18148	(0.7467)	1194	(0)	146	(0.2976)	25	(0.9222)	-8.7	(0.2162)	31	(0.2619)	-18.5	(0.2745)
20	Veneto	948871	(1)	19278	(0.5864)	2008	(0.7525)	825	(0.7796)	-6.7	(0.3964)	38	(0.0952)	-23.8	(0.0667)

## Questionnaire design

Information of interest was gathered from the respondents through a questionnaire (according to the policies of our ethical committee, ethical approval was not needed as the questionnaire was completely anonymous. The questionnaire was compliant with the Declaration of Helsinki as amended in 2013.) composed of 29 questions (see Appendix 2—Questionnaire). Its structure is divided into 2 main parts, which were functional to the respondent profiling and AHP methodology respectively. The first part aims at identifying the features of the sample, while the second session of questions aims at retrieving the information needed to construct the comparison matrices M, H, and E for each subject. The first part is comprised of 9 questions (Q1-Q9), the first five questions are essential to characterize the subject (i.e., gender, age) and its cultural background (i.e., level of education, profession); while the remaining four questions investigate the subject’s opinion on Covid-related issues that do not concern the health and economic criteria. In particular, the subjects are asked if they are willing to undergo the SARS-CoV-2 vaccine, whether they believed in a connection between COVID-19 and 5G technology, how their work position had changed after the disease outbreak, and which information sources they are accustomed to consult to keep updated on the status of the epidemic. In the second part (from question Q10 to question Q29), the subjects are asked a series of questions to assess the relative importance of one factor compared to another by weighing up all the criteria and sub-criteria in pairs. First, the subjects are asked for their personal opinion on the comparison between pairs of health healthcare sub-criteria (i.e., compiling matrix H) and then between pairs of economic sub-criteria separately (i.e., compiling matrix E). On the basis of these answers the comparison matrix of the health healthcare sub-criteria $H\in R^{4\times 4}$ and the comparison matrix of the economic sub-criteria $E\in R^{3\times 3}$ are built for each subject. In the end, each questionnaire respondent was asked to express a relevance weight for the comparison between the healthcare and economic criteria. This information is used to build matrix $M\in R^{2\times 2}$. Hence, for each subject, 3 matrices are available once the questionnaire has been filled in. Please note that the subject can compare the criteria or the sub-criteria with the following answers: “*significantly less important*”, “*a lot less important*”, “*slightly less important*”, “*equally important*”, “*slightly more important*”, “*a lot more important*”, “*significantly more important*” or leave the question blank. These textual answers have been translated into a corresponding numerical score of 1/7, 1/5, 1/3, 1, 3, 5, 7 respectively, according to Saaty’s scale, reported in Table 7. Note that when the subject did not provide an answer, a value of 0 was assumed, this means that the subject is not able to compare such a couple of criteria. To take into account this contingency, the ILLS methodology has been used. Before proceeding to the analysis of the results, we provide some considerations about the applicability of online surveys in such a research field. In the 1930s and 1940s, door-to-door interviews and mailing surveys were the two most popular ways to retrieve a large amount of data from people for various purposes. Since the 1970s, phone interviews became the most popular alternative. Nowadays, the Internet is increasingly present in our daily lives and web surveys are becoming an interesting option as well. Online surveys are generally considered cheaper, faster, and more convenient, especially in the pandemic context where social distancing was a limit in the information-gathering phase. Several aspects can affect the efficiency and validity of the proposed online survey. The first crucial point is the validity of the sampling of the population object of study. In general, the concept of validity is sharply defined for statistical studies. More precisely, there are two types of validity: external, and internal validity. Internal validity is defined as the extent to which the observed results represent the truth in the population we are studying and, thus, are not due to methodological errors. On the other side, external validity refers to the extent to which the results of a study can be generalized to other subjects, especially for the population that the sample is thought to represent. Usually, as mentioned in [58, 59], several measures are applicable to test the internal validity of a survey based on the AHP. The adoption of a consistency measure, such as the Consistency Ratio (CR) is one of the most useful approach to test the internal validity of this kind of study. Notice that, additional details about the internal validity are provided in the *Discussion* section. Concerning the external validity, according to the study presented in [60], external validity and generalizability are synonymous. In this view, a crucial check about the external validity can be set based on the results of the first part of the questionnaire. Is the sample representative of the population from which it was drawn? We remark that our questionnaire is composed of two parts, where the first one is essential to characterize the sample. As will be discussed in the following section, the main features of such a sample (e.g., the age distribution, the education qualification, etc) closely approximate the features of the sample of other statistical studies which involve a higher number of interviewed people. Hence, this encourages our research direction and the generalizability of the results.

## Description of the sample

**Fig 2:**
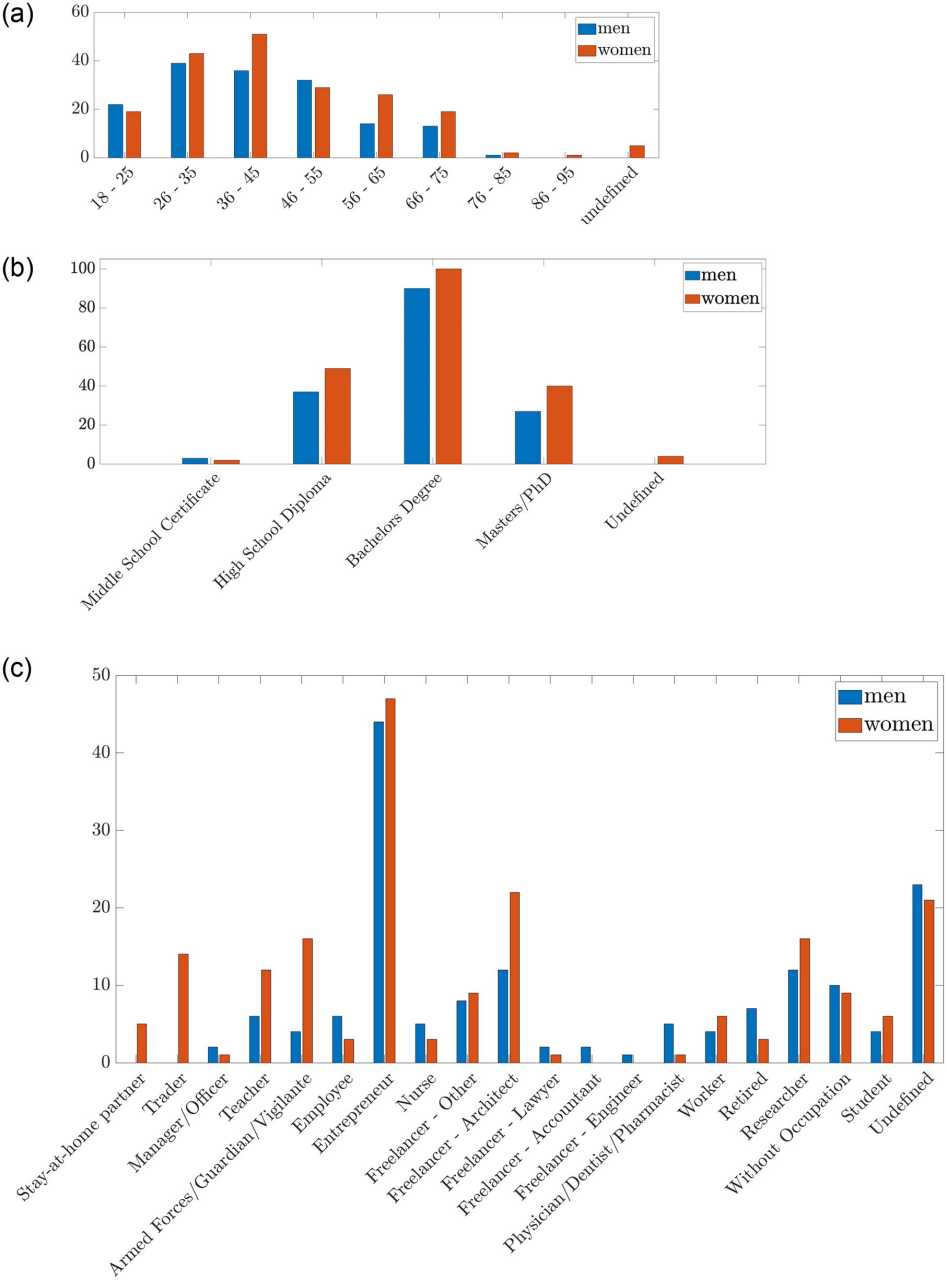
Characteristics of the sample. **(a)** Age Distribution, **(b)** Educational qualification held, **(c)** Occupation.

Based on the preliminary questions posed to the people surveyed (see Appendix 2—Questionnaire) in this section we present the description of the sample in its characteristic features. With reference to question Q2, the distribution of the 352 interviewed people among age-groups (Fig 2a) is as follows: 41 (12%) people belong to the age group 18-25, 82 (23%) to the age group 26-35, 87 (25%) in the age group 36-45, 61 (17%) to the age group 46-55, 40 (11%) to the age group 56-65, 32 (9%) to the age group 66-75, 3 (0.8%) to the age group 76-85, 1 (0.2%) to the age group 86-95, 5 (0.8%) is undefined (i.e., did not provide a answer to the age-related question).

With reference to question Q3, the distribution of the sample with respect to the educational qualification (Fig 2b) is as follows: 5 (1.4%) people have a Middle school certificate, 86 (24.4%) have a high school diploma, 190 (54%) have a Bachelor’s degree, 67 (19%) have a Ph.D. or M.Sc. degree, 4 (1.1%) is undefined (i.e., did not provide an answer). As mentioned in the previous section, the distribution of such characteristics of our sample represents a useful parameter to verify the external validity of the study. Notice that, the distribution about the age and the educational qualification closely approximates the characteristics of samples considered in other similar studies [61]. For the sake of precision, a small deviation between the features of our sample and the features of samples that involves a higher number of people is recognizable in the age and educational distribution. In our study the average age of the sample is slightly lower and, as a consequence, the medium level of education is slightly higher. Such difference could be attributed to the adoption of social networks as the main distribution channel for the online survey.

As described in Fig 2c, with reference to question Q5, the distribution of the interviewed people with respect to their occupation is: 5 (1, 4%) of people fall in the category stay-at-home partner, 14 (4%) in trader, 3 (0.8%) in manager/officer, 18 (5.1%) in Professor/teacher, 20 (5.7%) belong to Armed Forces/Guards/Vigilance, 9 (2.6%) in employee, 91 (25.9%) in entrepreneur, 8 (2.3%) in nurse, 17 (4.8%) in freelancer—other, 34 (9.7%) in freelancer—architect, 3 (0.8%) freelancer—lawyer, 2 (0.6%) in freelancer—accountant, 1 (0.3%) in freelancer—engineer, 6 (1.7%) in physician/dentist/pharmacist, 10 (2.8%) in factory worker, 10 (2.8%) in retired, 28 (8%) in researcher, 19(5.4%) in unemployed, 10 (2.8%) in student, 44 (12.5%) in undefined.

**Fig 3:**
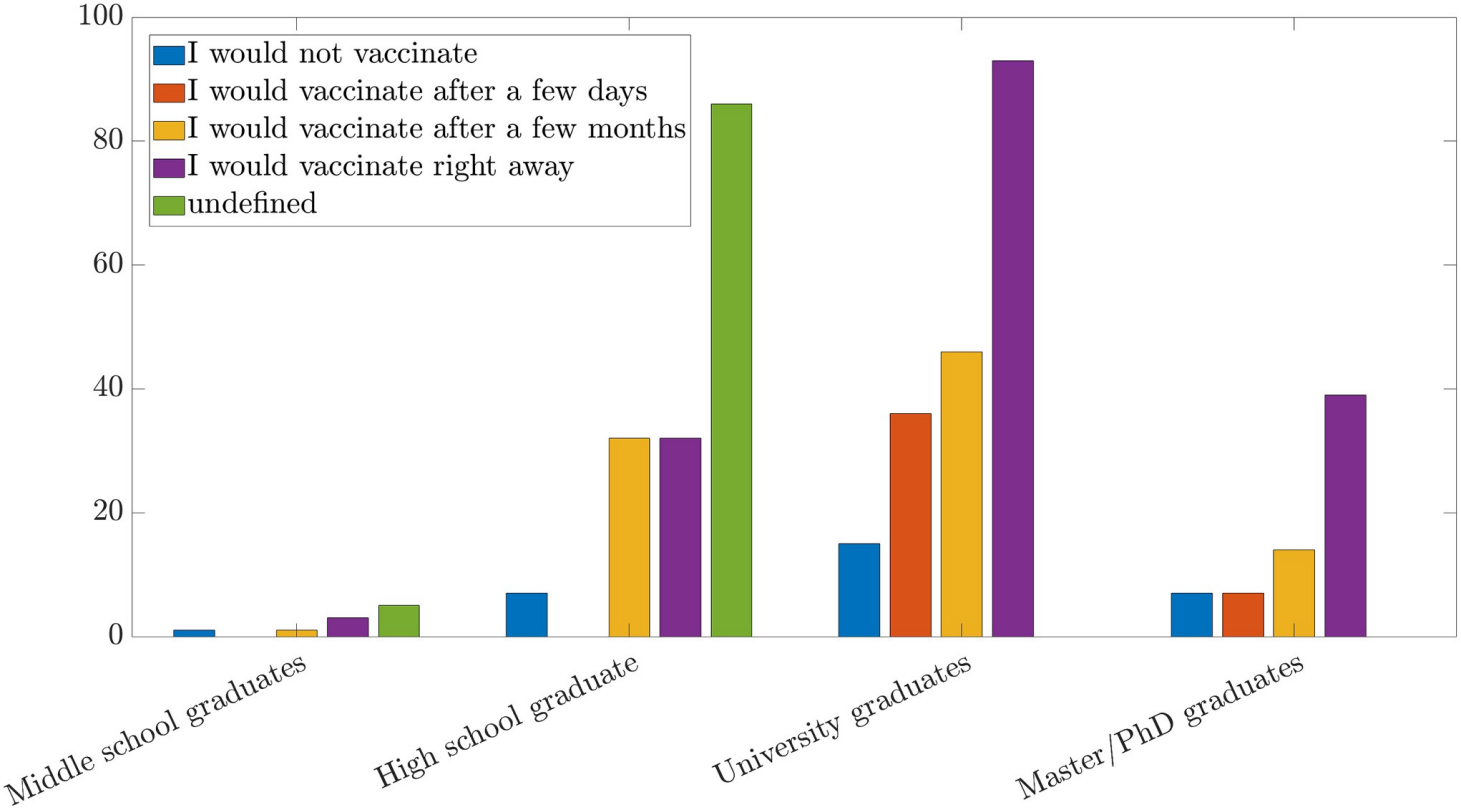
Propensity to vaccination for level of education.

From now on, we will discuss the propensity to vaccination (see question Q6) of the people surveyed with respect to different features of the sample (i.e., educational qualification, age groups). As depicted in Fig 3, it was found that, according to the educational qualification, the propensity to get vaccinated as soon as the vaccine would have been available, has the following pattern: 47% of people among all those with a University degree, 57% of people among those with a Ph.D. or M.Sc. degree, and this percentage drops to 38% if one considers people with a High School diploma. The data shows that the higher the level of education, the greater the propensity for immediate vaccination. Consequently, it can be deduced that 53% of university graduates, 43% of people with a Ph.D. or M.Sc. degree, and 62% of high-school graduates would wait, a few days to a few months, to get vaccinated. This trend may suggest greater confidence in research and in the reliability of new discoveries in people with higher educational qualifications. This could be related to a better capability to understand scientific jargon and studies, to an individual attitude to keep informed and constantly updated on the latest news as well as to select with awareness reliable sources of information. Indeed, it has emerged that the answer “Scientific Articles; Official Bulletins of Health Facilities; Reports of Statistical Survey” to the question on the source of information has been chosen predominantly by people with a Ph.D. or M.Sc. degree, while all the others have expressed their preference for TV news or newspapers. The latter ones are notoriously mass media, which already make a selection of the news to be broadcast as they are addressed to a very wide and diverse audience, as opposed to scientific journals that report more detailed news from a technical and scientific point of view and thus cannot be understood by everyone but they represent a more reliable tool of information, see Fig 3.

**Fig 4:**
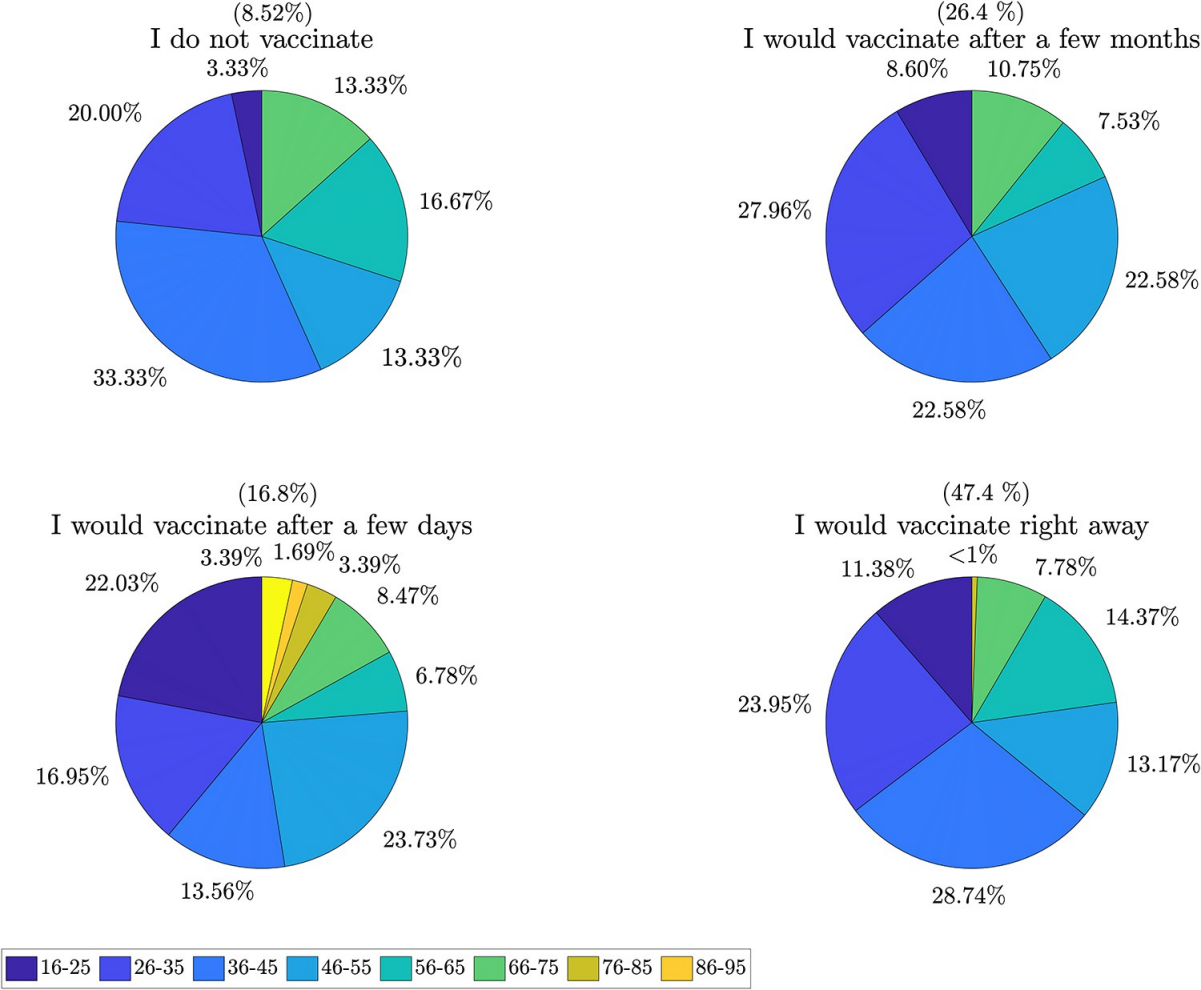
Analysis of the propensity to vaccination for group of age. Each pie chart reports the percentage of people per age group (represented in different colors, as shown in the legend) that agree to each of the following statements: “I do not vaccinate”, “I would vaccinate after a few months”, “I would vaccinate after a few days”, and “I would vaccinate right away”.

The propensity to vaccination (question Q6) was analyzed for the different age groups considered (Fig 4). It emerges that the age group most inclined to immediate vaccination is the 56-65 age group. This figure is certainly linked to the incidence of the disease in the over-60 age group, with a consequently higher mortality rate. On the contrary, the propensity to immediate vaccination in the 18-25 age group is 46%. This finding may be related to the fact that these individuals were less at risk of contracting the virus and having serious effects on their health. In addition, there were clear instructions from the national government to vaccinate the old and thus most fragile and at-risk individuals as a priority. The propensity to get immediately vaccinated in the 26-35 and 36-45 age groups is higher than in the youngest group, 49% and 54% respectively, probably due to the higher level of education found in these age groups, combined with a general state of health that poses no risk for possible adverse reactions to the vaccine. Lastly, in the 46-55 age group, the percentage of people willing to vaccinate immediately falls to 43%. This group realistically includes at-risk individuals or people with previous pathologies that could conflict with the effects of the vaccine. It should be noted that data for the even older age groups were not presented because of the small number of representative subjects enrolled in the study.

During the first lockdown in Italy, fake news was diffused claiming a correlation between the spread of the pandemic and 5G technology (see question Q7) [62, 63]. No scientific evidence was provided to support the claim, and scientific and health authorities denied the correlation. Yet, many citizens believed in such false statements, being informed by unreliable sources. For this reason, it was decided to conduct a preliminary analysis to understand how the sample may have been influenced in its choices and opinions by the spread of false or not entirely true news. In fact, there are many external factors that can affect the reliability of the highlighted sample. One of these factors is people’s lack of propensity for critical analysis and disability to not be swayed in their opinion by unreliable sources of information [64]; in general, emotional distress and education play a crucial role in consuming information from unchecked sources [11]. Thus, we found it interesting to evaluate people’s opinions about this theory and in particular the propensity to believe manifestly fake news in relation to the educational qualification level.

**Fig 5:**
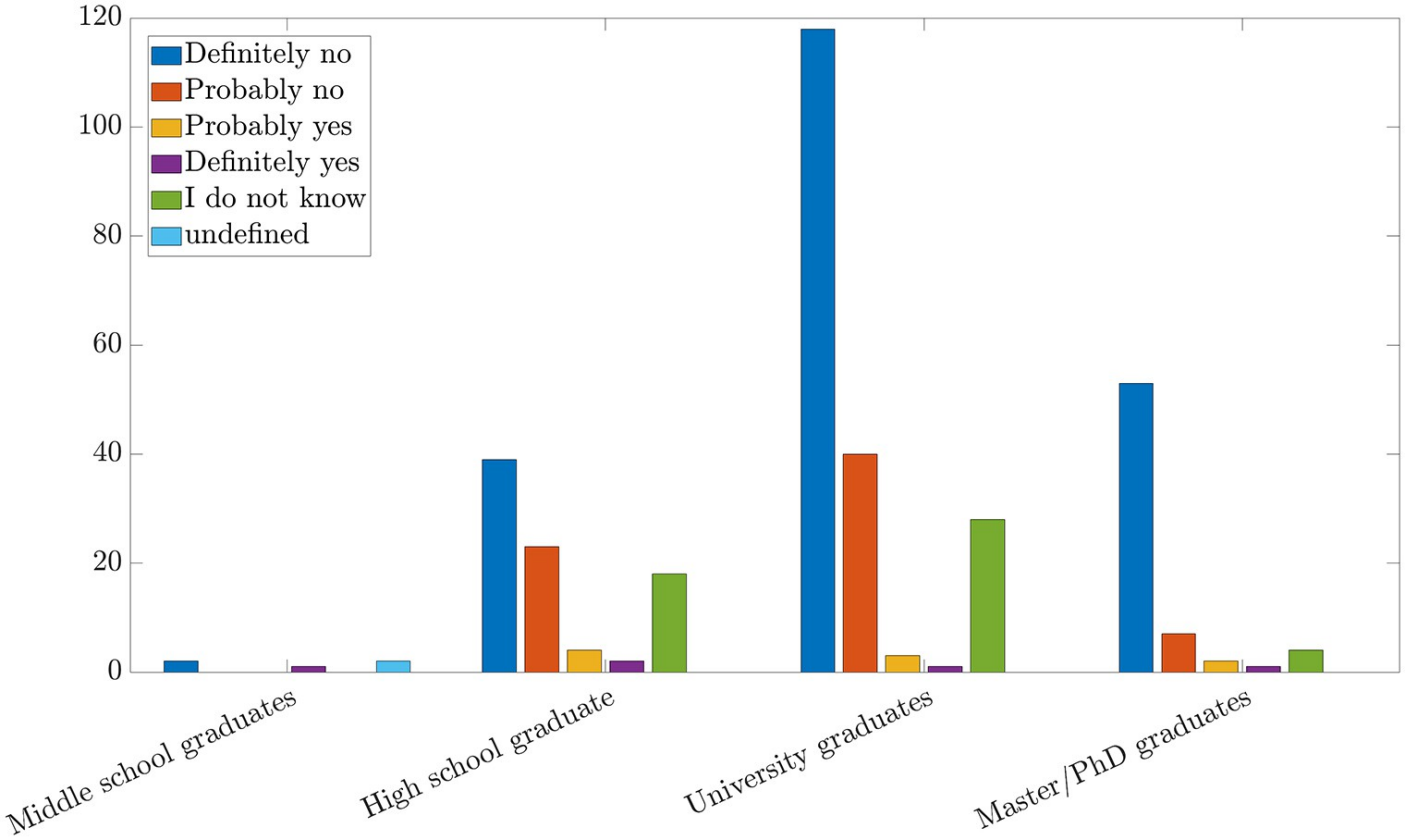
Relation between 5G and Covid-19 for level of education (express in absolute value).

As expected, the analysis showed that the propensity to believe this type of news decreases as the level of education increases (see Fig 5). Furthermore, one can support this theory by evaluating the sources of information that the subjects read. Indeed, people with a higher educational qualification tend to consult more reliable and technical sources of information like scientific journals so they are less likely to believe fake news because they have the tools to perform a critical analysis and a discernment based on technical-scientific concepts.

## Results

In this section we provide the most relevant aspects of our research about the evaluation of the regional policies to contrast the spread of COVID-19. A first general discussion about such policies is provided in the section “General Results” where the entire interviewed population is considered in the evaluation process. Two additional interesting outcomes of our research are summarized in the sections *Regional Policies evaluation in 18-25 age group* and *Relationships between the reluctance to get vaccinated and the evaluation of regional policies*. Such results describe the relations between a subset of the interviewed population characterized by particular features and their opinions in the evaluation process.

### General results

In this subsection, we propose an overall evaluation of the regional policies considering the entire interviewed population. Notice that in the first part of this analysis, we provide the details about the estimation of the weights (see Appendix), while the second part provides the ranking of the regional policies on the basis of the computed weights.

#### Local and global weights estimation

As mentioned in the section *Proposed Evaluation Scheme*, the preliminary step for the evaluation of the Italian regions’ response to the pandemic emergency is the computation of the weights associated with each level of the hierarchical structure depicted in Fig 1. In more detail, on the basis of the questions Q10 and Q11, we identify the criteria weights **m**_1_ and **m**_2_ as the solution **y** of the optimization problem defined by Eq (3). More precisely, we solve such a problem by considering *g* = 352 and *n* = 2, while the comparison matrices *W*^(*u*)^ correspond to the comparison matrices $M^{\left(u\right)}$ defined on the basis of questions Q10 and Q11 for each questionnaire respondent *u*. Similarly, the same approach based on the solution of the optimization problem (see Eq (3) is used to compute the values of the local weights $h_{1},\ldots ,h_{4}$ associated to the healthcare criteria, and the local weights $e_{1},\ldots ,e_{3}$ associated to the economic criteria. In more detail, the vector h corresponds to the solution of the same optimization problem considering *g* = 352 and *n* = 4, while the comparison matrices *W*^(*u*)^ correspond to the comparison matrices $H^{\left(u\right)}$ defined on the basis of questions Q12 and Q23 for each questionnaire respondent *u*. Similarly, the vector e, which represents the collection of the weights associated with the economic criteria, corresponds to the solution of the same optimization problem considering *g* = 352 and *n* = 3, while the comparison matrices *W*^(*u*)^ correspond to the comparison matrices $E^{\left(u\right)}$ defined on the basis of questions Q24 and Q29 for each questionnaire respondent *u*. The numerical values associated with the weights are summarized in Table 3.

**Table 3:** Local and global weights for criteria and sub-criteria.

Criteria local weights (**m**)	Sub-criteria description	Sub-Criteria Local weights	Global weights (*w*_*i*_)
Healthcare	Number of COVID-19 molecular tests	**h**_1_ = 0.2683	0.1712
0.6382	Number of COVID-19 cases	**h**_2_ = 0.2433	0.1553
	Number of deaths due to COVID-19 consequences	**h**_3_ = 0.1902	0.1214
	Number of ICU beds	**h**_4_ = 0.2981	0.1903
Economic	Companies lost revenue	**e**_1_ = 0.3060	0.1107
0.3618	Number of suspended workers	**e**_2_ = 3667	0.1327
	Impact on tourism and transport supply chain	**e**_3_ = 3273	0.1184

For what concerns the comparison between the criteria, it emerges that the healthcare criteria have a greater overall weight than the economic criteria, in fact, the former have a local weight of 63.82% while the economic criteria have a local weight of 36.18%. It can be concluded that there is a higher sensitivity of the people surveyed to the health needs affecting Italy rather than to the adverse economic conditions.

The general ranking of the local weights has been established both for the healthcare parameters and economical sub-criteria. In particular, considering the healthcare sub-criteria, it can be seen that the parameter with the greatest relevance is the *number of beds in intensive care*, which has a local weight of 29.81%, followed by the *number of COVID-19 molecular tests* (26.83%), the *number of COVID-19 cases* (24.33%) and finally the *number of deaths due to COVID-19 consequences* (19.02%). Surely there is a strong inter-correlation among the different sub-criteria; however, according to popular opinion, it emerges that more intensive ICU places and more swabs performed would have made it possible to avoid such a high number of deaths. Moreover, performing more swabs would have allowed earlier detection of positive cases to isolate them and reduce the overall number of cases (see Table 1).

With regard to the economic sub-criteria, according to the interviewed sample, the parameter which had the greatest local weight is the *number of suspended workers* (36.67%), followed by the *impact on the tourism and transport supply chain* (32.73%) and by the *lost business turnover* with a weight of 30.60%, see Table 1. As with the health sub-criteria, the impact that the pandemic has had on the tourism and transportation sector has led to consequences both in terms of lost business revenue for companies and the suspension of workers. Hence the discrepancy in weight between the different criteria. Then the impact on the tourism and transport supply chain has greater weight because Italy is a country that benefits from a high flow of tourists all year round and has based much of its business on this sector.

Analyzing all the global weights (*w*_*i*_), the general ranking of importance, on the basis of the whole sample, is the following: *ICU places* (19.03%), *number of COVID-19 molecular tests* (17.12%), *Number of COVID-19 cases* (15.53%), *number of suspended workers* (13.27%), *number of deaths due to COVID-19 consequences* (12.14%), *impact on tourism and transport supply chain* (11.84%), *companies lost revenue* (11.07%). These global weights have been obtained by combining the sub-criteria local weights (*h*_*i*_ or *e*_*i*_) expressed for the healthcare and economic sub-criteria with the criteria local weights (*m*_*i*_). Obviously, even in the general ranking the healthcare sub-criteria still have higher weights than economic ones.

#### Italian regions evaluations

On the basis of the global weights computed as mentioned in the previous section, we now provide an overall evaluation, based on Eq (1), of each Italian region considering its ability to counter the COVID-19 pandemic.

**Fig 6:**
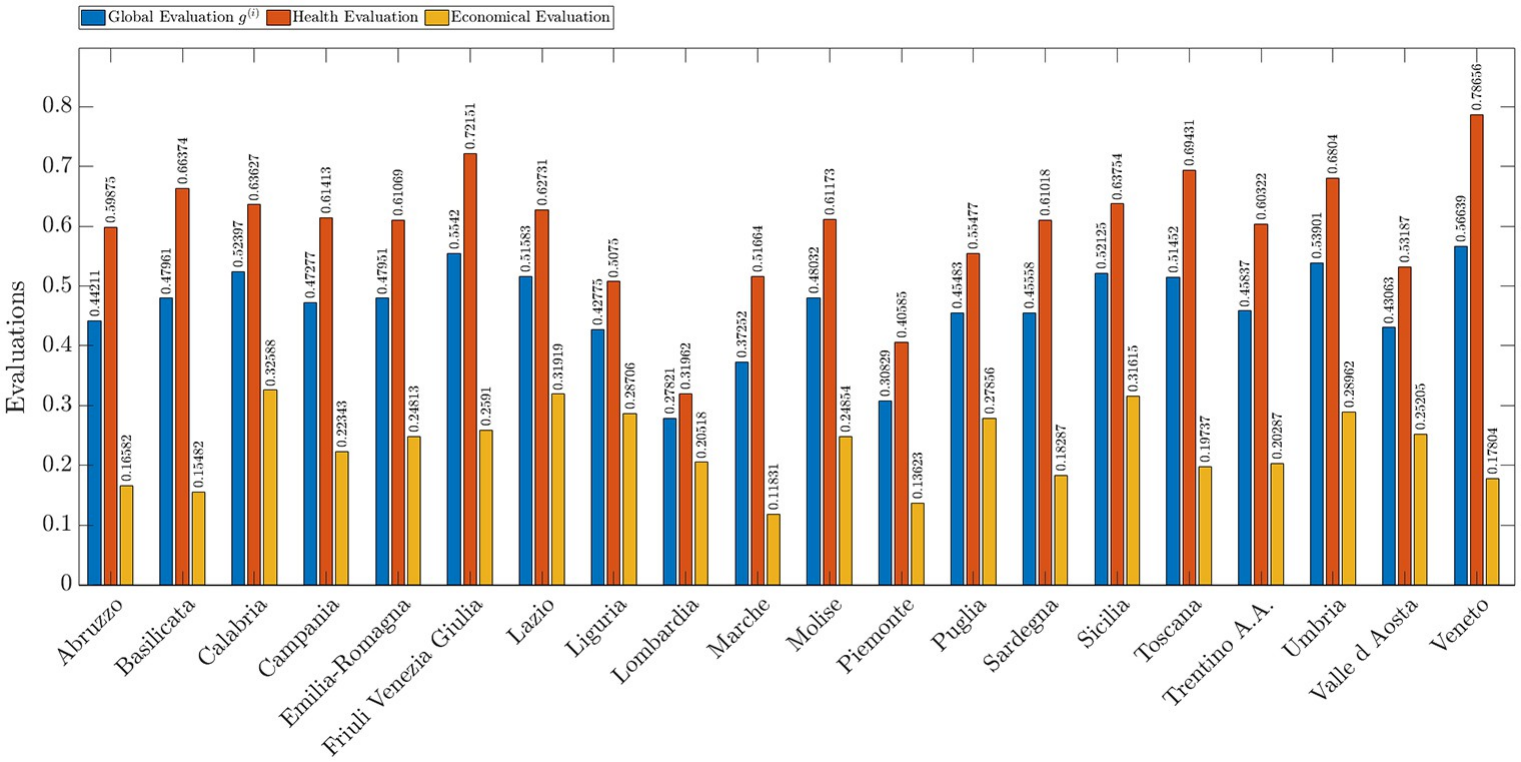
Overall evaluation (blue), and partial evaluations based on economic (green) and healthcare criteria (red) for Italian regions.

Fig 6 shows by means of blue bars the overall evaluations *g*^(*i*)^ that represent the ability to counteract the spread of COVID-19 of the *i*-th Italian region calculated according to Eq (1). In the same figure, we also provide by means of red and green bars the partial evaluation related to the healthcare and economic criteria respectively.

The three regions in which healthcare criteria have a higher evaluation are Veneto, Friuli Venezia Giulia, and Toscana. Instead, the region where the economic criteria have the higher evaluation are Lazio, Sicilia e Calabria.

In general, health evaluation carry more weight than economic evaluation in all regions. This undoubtedly comes from the uncertainty and fear of one’s own health. The pandemic event has disrupted everyone’s lives, and unpreparedness from a health perspective has resulted in the loss of many lives. In the face of this scenario, in this first phase of pandemic management, economic aspects were less prominent.

Note that when it comes to the evaluation of economic and health parameters, both the opinions of the subjects participating in the study and the objective data defining the different criteria are taken into account. For this reason, Lombardia and Piemonte, two of the first regions affected by the epidemic, were immediately affected by the closure of many companies in the area, the loss of labor caused by the high death rate, and the strict confinement measures imposed by the government, such as the inability to move from home except for proven emergency reasons, and for this reason, they have lower ratings on the economic parameters than other less industrialized regions. At the same time, low health ratings depend on unpreparedness in emergency management and the resulting high number of deaths.

In contrast, Veneto appears to have the highest evaluation of health parameters, although it is one of the most affected regions for a number of absolute cases. This is because Veneto immediately took action in countering the pandemic by subjecting the population to molecular testing; in fact, it turns out to be the region that has performed the most. In addition, having an adequate number of intensive care beds for the population has allowed for fewer deaths than in other regions.

The region with the worst evaluation with respect to economic parameters is Marche. This is due to the impact the pandemic has had on tourism and the transportation system. Marche has the highest number of suspended jobs and one of the highest values of impact on the tourism sector. These two aspects, combined with the significance of the weights provided for the economic criteria, cause Marche to have a very low rating for economic factors.

At the same time, two regions such as Sicilia and Calabria with a predominantly tourism-based economy were among the regions with the best ratings for economic factors. This is probably due to a series of economic aids that the Italian government provided prior to the pandemic events for citizens with economic difficulties, making them less affected by the closures imposed to contain the pandemic.

**Fig 7:**
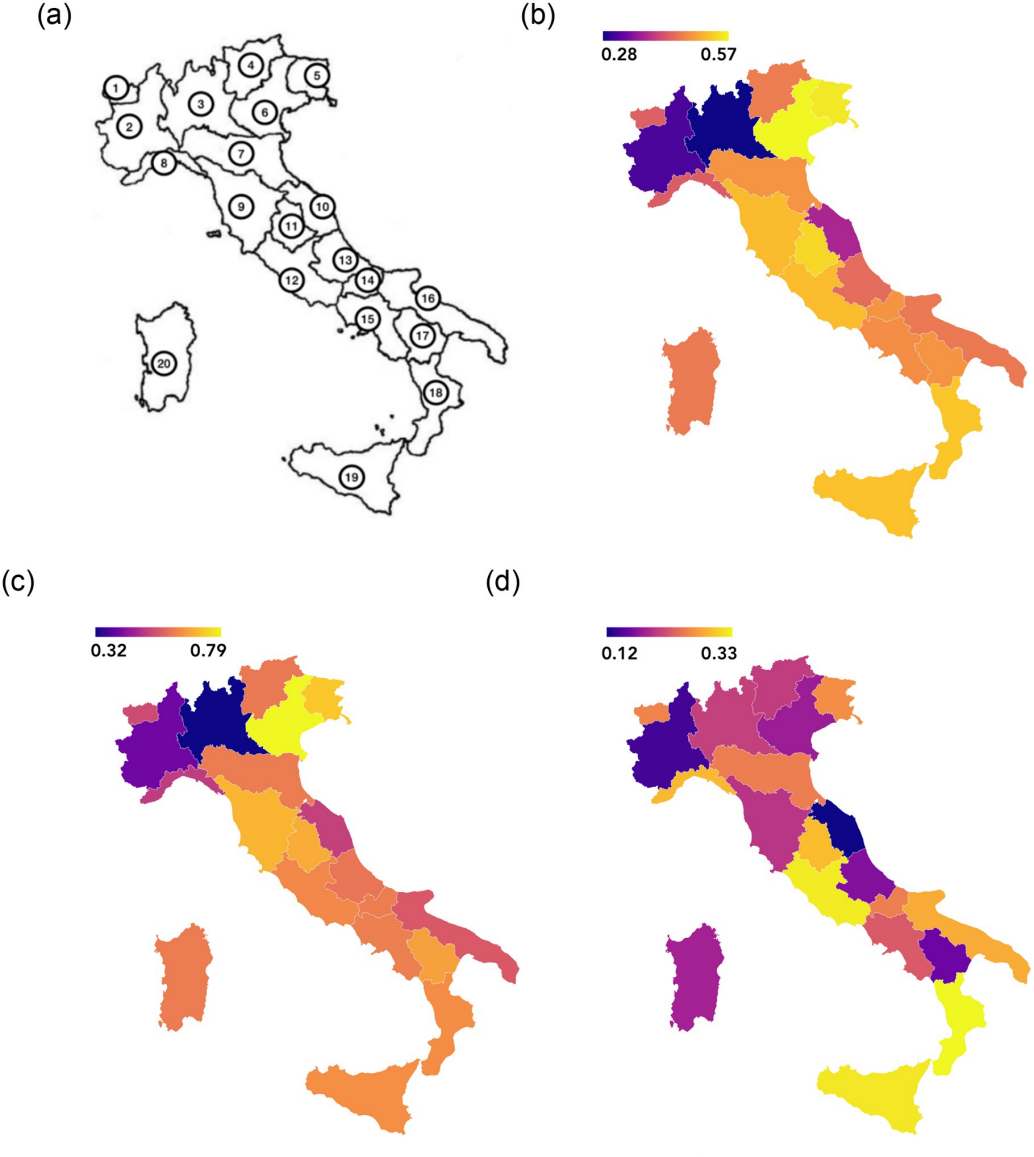
Italian regions evaluations. Light colors correspond to a high (positive) evaluation, dark colors correspond to a low (negative) evaluation. The regions are identified according to the following numbers: (1) Valle d–Aosta, (2) Piemonte, (3) Lombardia, (4) Trentino Alto Adige, (5) Friuli Venezia Giulia, (6) Veneto, (7) Emilia Romagna, (8) Liguria, (9) Toscana, (10) Marche, (11) Umbria, (12) Lazio, (13) Abruzzo, (14) Molise, (15) Campania, (16) Puglia, (17) Basilicata, (18) Calabria, (19) Sicilia, (20) Sardegna. **(a)** Map of the Italian regions, **(b)** Overall evaluation, **(c)** Partial evaluation based on healthcare criteria, **(d)** Partial evaluation based on economic criteria.

We conclude this first results overview by considering relations between the geographical position of each region and the results of the evaluation process based on the AHP. Fig 7 collects the results of our study considering the overall evaluation (Fig 7b), a partial evaluation based only on the healthcare criteria (Fig 7b), and a partial evaluation based only on the economic criteria (Fig 7b). Such a figure proposes the same results as the bar plot in Fig 6 with a geographical perspective. According to such representation, we can conclude that considering the overall evaluation, the northern regions have coped worse with the emergence due to the pandemic, with the exception of Veneto which, as mentioned above, obtained a high evaluation according to the healthcare criteria.

### Regional policies evaluation in 18-25 age group

**Fig 8:**
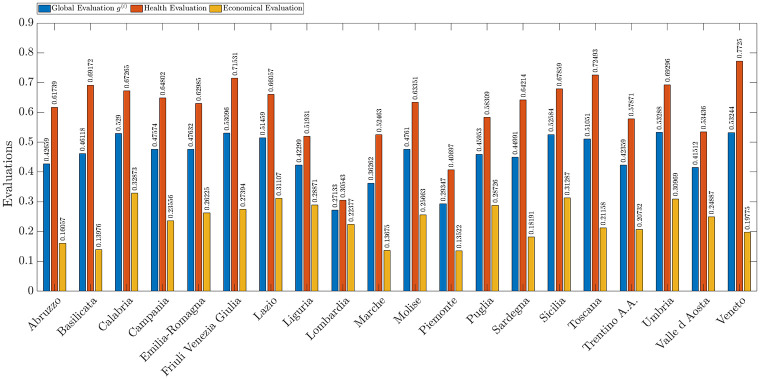
Overall evaluation (blue), and partial evaluations based on economic (green) and healthcare criteria (red) for Italian regions considering only respondents in the 18-25 age group.

In this section, we consider only the respondents in the 18-25 age group (41 people interviewed). According to Fig 8, we can state that, even if we limit the study to this particular age group, the overall regional evaluations does not change significantly. In general, it turns out that despite different age groups there is no different view of the relevance of healthcare and economic aspects in the evaluation process. This is probably because health concerns different segments of the population indiscriminately.

As shown in Table 4, the only substantial difference in the results of this age group with respect to the general case is related to the relevance of the two criteria. Similarly to the general case, we observe greater importance of health criteria than economic criteria, but in this case, the gap between the criteria weights is reduced. This is probably due to the uncertainty that this age group sees in its future.

**Table 4:** Local and global weights for criteria and sub-criteria considering the 18-25 age group.

Criteria weights (**m**)	Sub-criteria description	Local weights	Global weights (*w*_*i*_)
Healthcare	Number of COVID-19 molecular tests	**h**_1_ = 0.1888	0.1100
0.5823	Number of COVID-19 cases	**h**_2_ = 0.2176	0.1267
	Number of deaths due to COVID-19 consequences	**h**_3_ = 0.2466	0.1436
	Number of ICU beds	**h**_4_ = 0.3470	0.2021
Economic	Companies lost revenue	**e**_1_ = 0.3693	0.1542
0.4177	Number of suspended workers	**e**_2_ = 0.3261	0.1362
	Impact on tourism and transport supply chain	**e**_2_ = 0.3046	0.1272

We now compare the healthcare local weights and then the economic local weights of this subset of individuals with those obtained considering the whole sample. It can be seen that the *number of COVID-19 molecular tests* has a weight of 0.1888 while in the general sample, it was 0.2683, while the parameter *number of COVID-19 cases* changes from 0.2433 to 0.2176 and *number of deaths due to the consequences of COVID-19* was 0.1902 and becomes 0.2466, finally the *number of ICU places* becomes 0.3470 from 0.2981.

It emerges that in this age group, the most relevant parameter is the number of ICU beds instead of the number of pads, which has become the least relevant parameter. There is also a reversal of relevance between the number of cases and the number of deaths, which becomes the second most relevant parameter. These data can be explained by the incidence that COVID-19 had in this age group. Young boys seemed to be less affected by the disease but saw more frail and older people dying or unable to access treatment. Undoubtedly this may prompt consideration of the parameters of greater the number of places in intensive care and the number of deaths. This aspect is once again congruent with the critical health management that took place in the country during the emergency, where there were insufficient ICU places throughout the entire territory. This lack led to the need to build and install new intensive care units in several hospitals throughout the country. This operation took a relatively long time leading to the loss of numerous lives in the meantime.

The weights assigned to the various economic local weights, according to the different age groups, are now analyzed. In this case, the overall trend of the criteria does not change much but the most relevant criterion varies. The most relevant criterion is *companies lost revenue* with a weight of 0.3693 followed by *suspend workers* with 0.3261 and *impact on tourism and transport supply chain* with 0.3046. This figure is interesting because it probably also affects the evaluation of the others since a loss of turnover means a reduction in the funds available to the companies and the consequent need to make staff cuts. It must be kept in mind that this segment of the population is the least likely to have been employed but rather is predominantly composed of students.

Considering only the health parameters in the evaluation process, the best evaluation appears to be the Veneto for the general case albeit having a reduction in value. Despite thus having a worse evaluation, in this subgroup, it has the best evaluation compared to the others. Similarly, Lombardia still turns out to be the region with the worst health evaluation, again there is a reduction in the resulting value. Notably, a reduction in the rating of health parameters also occurs for Friuli Venezia Giulia, which, however, remains one of the regions with the highest rating for health parameters.

Now analyzing only the economic parameters, the region with the best rating in the 18-25 age group is Calabria with a value of 0.32873, followed by Lazio with 0.31107 and Sicily with 0.31287. Thus, the trend that was presented when considering the entire sample is confirmed. In this case, albeit by a small margin, the region with the worst management of economic parameters turns out to be Piemonte with 0.1352, narrowly preceded by Marche with 0.1368. The latter region confirms the worst management of economic parameters although considering the sample under review presents a higher rating than the full sample. In addition, there are other regions such as Molise, Puglia, Toscana, Umbria, Valle d’Aosta, and Veneto that have a higher evaluation of economic parameters than the full sample. This is given by the higher consideration of the subjects under study for economic parameters.

### Relationships between the reluctance to get vaccinated and the evaluation of regional policies

**Fig 9:**
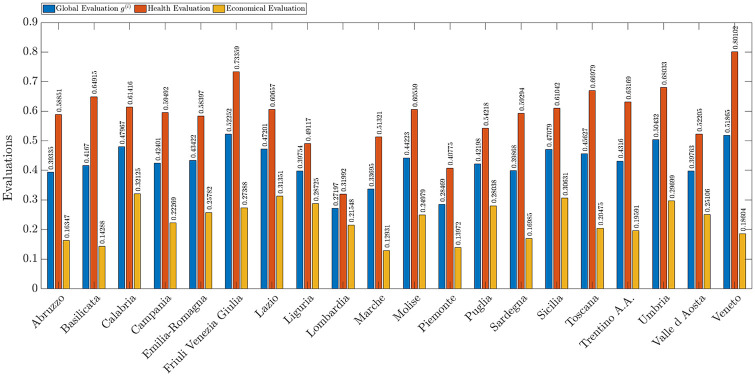
Overall evaluation (blue), and partial evaluations based on economic (green) and healthcare criteria (red) for Italian regions considering only people who would not vaccinate.

The relevance of healthcare and economic parameters with respect to reluctance to get vaccinated is now analyzed. It was intended to understand whether there was less or more attention to healthcare or economic issues despite the reluctance to get vaccinated. In general, the result in terms of regional ranking does not show substantial changes with respect to global evaluations. In Table 5 we show the criteria weights, the local weights, and the global weights obtained considering only the subset of people surveyed characterized by a reluctance to get vaccinated (30 people), while in Fig 9 we show the overall evaluation of each region and the partial evaluations based on the health and the economic criteria.

**Table 5:** Local and global weights for criteria and sub-criteria considering the group of people that would not vaccinate.

Criteria weights (**m**)	Sub-criteria description	Local weights	Global weights (*w*_*i*_)
Healthcare	Number of COVID-19 test	**h**_1_ = 0.3374	0.1825
0.5409	Number of COVID-19 molecular cases	**h**_2_ = 0.2490	0.1347
	Number of deaths due to COVID-19 consequences	**h**_3_ = 0.1781	0.0963
	Number of ICU beds	**h**_4_ = 0.2355	0.1274
Economic	Companies lost revenue	**e**_1_ = 0.3351	0.1539
0.4591	Number of suspended workers	**e**_2_ = 0.3103	0.1425
	Impact on tourism and transport supply chain	**e**_3_ = 0.3546	0.1628

For this group of people, the relevance of the healthcare criteria is reduced compared to the global trend and the 18-25 age group (see Tables 3 and 4 respectively), moreover, the economic aspects assume greater importance. In particular, the healthcare criteria have a weight of 0.5409 and the economic criteria have a weight of 0.4591.

This trend can be also noted by analyzing the obtained global weights. The most relevant criterion is *the number of COVID-19 tests* (0.1825), followed by the *impact on tourism and transport supply chain* (0.1628) and *Companies lost revenue* (0.1539). These findings demonstrate that people who showed an aversion to vaccination, have less sensitivity to health-related issues and consequently show a greater consideration of the economic aspects. In fact, these people believed that all the restrictive measures taken by the various regions to contain the spread of contagions (e.g. local lockdowns) would have had a too heavy impact on business from an economic point of view.

Although this change in the general trend of the criteria evaluation, the best-evaluated regions, considering the healthcare criteria, are still Veneto, Friuli Venezia Giulia, Umbria, and Toscana, with a slight variation in their values. In particular, Veneto and Friuli Venezia Giulia show an increase in their evaluation while Toscana has a decrease in its weight. From the economic point of view, the first regions in the general ranking are Calabria, Lazio, and Sicilia, the same regions as in the general ranking for the global sample, except for Sicilia which has a slightly lower evaluation.

## Discussion

The aim of our study was to assess how the COVID-19 management policies undertaken during the first stages of the pandemic of the various Italian regions were welcomed and assessed by the residents, on the basis of healthcare and economic criteria. The evaluation was performed through a survey provided to 352 individuals from different regions. Notice that the use of a questionnaire that includes both questions for the interviewee’s profiling and questions relating to the relative evaluations necessary for the application of the AHP allows searching for interesting correlations between the perception of the goodness of a regional policy and anthropological and cultural aspects of the population. Notice further that, on the one hand, the use of a questionnaire does not allow for additional explanations to be provided to the interviewees, on the other hand, it allows for a sufficiently representative sample to be reached even during the pandemic. From the collected data we computed the overall ranking of the Italian regions with respect to economic and healthcare criteria using the AHP technique. Moreover, we showed the relevance weights of these criteria and specific aspects (i.e., sub-criteria) for the sample considered. Results were presented showing the importance weights of the healthcare and economic criteria and the associated sub-criteria. Moreover, it was analyzed how these results change by filtering the answers of a part of the sample with specific features (e.g., age groups, level of education). Furthermore, a preliminary analysis was made on the composition of the sample to assess the internal (e.g., age, the status of employment, cultural background) and external influences on the subjects’ judgment (e.g., source of information, fake news) that completed the questionnaire: to better understand their stance on issues such as the propensity to get vaccinated and susceptibility to fake news spread by the mass media. The analysis of the results shows that Veneto is the region whose policy to contrast the spread of COVID-19 was perceived as the most effective. This result is due to the high attention of the region in carrying out molecular tests for the detection of the virus and great attention to the strengthening of the health infrastructure. Overall, the results discussed are verified through our methodology for most of the possible subsets of the surveyed population (e.g., considering occupation, education, etc.). The first exception is the 18-25 age group population subset. In this case, the difference between the weight of health and economic criteria is significantly reduced in the regional policy evaluation process. However, the health sub-criteria of this subset shows a high sensitivity to the incidence of deaths caused by COVID-19 in the oldest segments of the population and the frailest. A further reduction in the difference between the weight of health and economic criteria in the regional policy evaluation process can be observed in the subset of respondents least inclined to vaccination. In this subset, it also appears that the factor related to the number of COVID-19 deaths in evaluating regional policies loses importance. Concerning the applicability of the approach, thanks to the adoption of the hierarchical structure of the approach, the number of relative judgments required to the interviewed subjects is reduced from 21 to 9 comparisons. In addition, such approach admits incomplete information.

We conclude this section with a critical review of the proposed approach. Surely, our approach has the classic limits related to the decision-making approaches based on pairwise comparisons. Since it is necessary to compare the individual criteria and then compare the alternatives with respect to each specific criterion, the decision-making process can be time-consuming and computationally intensive. Notice that, in this study, we adopt the sparse version of AHP in order to reduce the inconsistency and the time required to provide the relative evaluations. Concerning the problem related to the inconsistency, in this study we require to provide relative judgments (from Q10 to Q29) considering a small number of criteria. Notice that the largest pairwise comparison matrix is H which encompasses the relative judgments among four criteria. The small number of considered criteria limits the subject in introducing inconsistency during the filling process. For the sake of completeness, according to the definition of judgments inconsistency (see Appendix 1—Analytic Hierarchy Process), we summarize in Table 6 the average value of the inconsistency (in terms of Consistency Ratio) and the number of consistent and inconsistent comparison matrices considering the matrices related to the health (H) and economic (E) sub-criteria. As summarized in Table 6, the average inconsistency level can be considered acceptable for the largest part of the instances provided by the interviewees. Notice that such values of inconsistency are a valid result in terms of internal validity, as mentioned in section *Questionnaire Design*. Another limitation of this study is that the AHP methodology does not take into account the inter-correlation between the considered criteria. For instance, with regard to the healthcare criteria, the number of ICU places is a factor that has definitely influenced the number of deaths, while the number of cases reported is highly dependent on the number of molecular swabs that were taken. Similarly, for what concerns the economic criteria, the change in the job position of surveyed people may have been dependent on the loss of capital from the belonging Enterprises. There are other techniques, such as the ANP method, which take into account the correlation of parameters in the evaluation but this approach calls for a methodology able to identify a quantitative evaluation able to better represent such correlations. In addition, for future studies, we would like to extend this work to a larger number of respondents to take into account a wider sample of the population and also extend the work to a longer time frame that takes into account the final phase of the pandemic and allows us to evaluate regional policies based on the management of the entire phenomenon and of its consequences.

**Table 6:** Local and global weights for criteria and sub-criteria considering the group of people that would not vaccinate.

Comparison Matrix	Average Inconsistency $\left(\tilde{CR}\right)$	Consistent Instances	Inconsistent Instances
E	0.0215	350	2
H	0.0373	329	23

## Conclusions

Our study aimed at assessing how the COVID-19 management policies of the different Italian regions were evaluated by the citizens on the basis of healthcare and economic criteria at the beginning of this natural calamity. To pursue this objective, a survey was designed to gather responses from the population using 29 items. The collected data were analyzed using the AHP methodology. In the region evaluation process, results showed that in general, the healthcare criteria have a greater relevance (with a weight of 63%) than the economic criteria (36%). In more detail, the global weight of each sub-criteria in the overall evaluation process is the following *ICU places* (19.03%), *number of COVID-19 molecular tests* (17.12%), *Number of COVID-19 cases* (15.53%), *number of suspended workers* (13.27%), *number of deaths due to COVID-19 consequences* (12.14%), *impact on tourism and transport supply chain* (11.84%), *companies lost revenue* (11.07%). It can be concluded that this result confirms a higher sensitivity of people surveyed to healthcare issues rather than to adverse economic conditions. These findings suggest that despite a severely compromised national economic condition, the healthcare-related problems have been perceived as highly relevant in the management of the COVID-19 emergency. For further developments, we intend to extend the pool of subjects and the criteria analyzed and to collect more recent data to compare how popular opinion changed over time with respect to the increased familiarity with the effects of the pandemic and its management. Another future research direction will consider the inter-dependencies within the sets of criteria and sub-criteria providing a new framework based on the Analytic Network Process. Moreover, we want to apply a similar framework to the problem related to the “long covid syndrome” by evaluating the relevance of the most frequent symptoms that affect recovered patients.

## Appendix

### Appendix 1—Preliminaries

#### Notation

We denote vectors by boldface lowercase letters and matrices with uppercase letters and we refer to the (*i*, *j*)-th entry of a generic matrix *A* by *A*_*ij*_. We represent by **1**_*n*_ the column vector with *n* components all equal to one.

#### Graph-theoretical preliminaries

Let *G* = {*V*, *E*} be a *graph* with |*V*| = *n* nodes *V* = {*v*_1_, *v*_2_, …, *v*_*n*_} and *e* = |*E*| edges *E* ⊆ *V* × *V* where (*v*_*i*_, *v*_*j*_) ∈ *E* represents the existence of a link from node *v*_*i*_ to node *v*_*j*_. A graph *G* is *undirected* if (*v*_*i*_, *v*_*j*_) ∈ *E* whenever (*v*_*j*_, *v*_*i*_)∈*E* and is *directed* otherwise. Let *G* be an undirected graph, the *neighborhood* $N_{i}$ of a node *v*_*i*_ is the set of nodes *v*_*j*_, such that (*v*_*i*_, *v*_*j*_)∈*E*. For a given node *v*_*i*_, the *degree* *d*_*i*_ of the node is the number of edges incident on it; i.e., $d_{i}=\left|N_{i}\right|$. Given a graph G = {V, E} with *n* nodes, we define the *Laplacian matrix* L as the *n* × *n* matrix such that

$$\begin{array}{c} L_{ij}=\{\begin{array}{cc} d_{i}, & \text{if}\;i=j\\ -1, & \text{if}\;(v_{i},v_{j})\in E\\ 0, & \text{otherwise} \end{array} \end{array}$$

Note that the *Laplacian matrix* L *can also be expressed as*:

$$\begin{array}{c} L=D-A \end{array}$$

*where* A *is the Adjacency matrix*:

$$\begin{array}{c} A_{ij}=\{\begin{array}{cc} 1, & \text{if}\;(v_{i},v_{j})\in E\\ 0, & \text{otherwise} \end{array} \end{array}$$

that expresses whether a link between the i-th and j-th node is present, and D is the *Inverse Degree matrix*:

$$\begin{array}{c} D_{ij}=\{\begin{array}{cc} d_{i}, & \text{if}\;i=j\\ 0, & \text{otherwise} \end{array} \end{array}$$

that keeps trace of the degrees of the n nodes constituting the graph.

#### Analytic Hierarchy Process

The Analytic Hierarchy Process (AHP) is an accurate tool for decision-making based on multiple criteria, developed by Thomas L. Saaty in the 1970s [13]. AHP finds application in many fields and it is particularly useful when dealing with complex problems in which the elements of the decision are difficult to quantify or compare [17].

The AHP approach exploits human relative judgments for quantifying the absolute evaluations or weights of the multiple decision criteria and can be conceptually summarized into the following steps:

1. The decision problem is decomposed into a hierarchy of simpler sub-problems that can be analyzed independently.
2. Decision makers evaluate the elements of the hierarchy by comparing them to each other in pairs.
3. AHP converts these relative evaluations into absolute numerical values and a numerical weight is derived for each element of the hierarchy, allowing a comparison between diverse events in a consistent way.
4. Numerical priorities are calculated for each of the decision alternatives.

The aim of the AHP approach is the evaluation of a set of *p* alternatives considering multiple criteria and sub-criteria organized considering a hierarchical approach. Each criterion (or sub-criterion) is associated with an unknown positive weight *w*_*i*_ > 0, which expresses its relevance. Although the absolute value of *w*_*i*_ is not known, it is possible to build a pairwise comparison matrix, containing the relative importance of each pair of criteria (or sub-criteria) weights:

$$\begin{array}{c} W=[\begin{array}{cccc} \frac{w_{1}}{w_{1}} & \frac{w_{1}}{w_{2}} & \ldots & \frac{w_{1}}{w_{n}}\\ \frac{w_{2}}{w_{1}} & \frac{w_{2}}{w_{2}} & \ldots & \frac{w_{2}}{w_{n}}\\ & & \ddots \\ \frac{w_{n}}{w_{1}} & \frac{w_{n}}{w_{2}} & \ldots & \frac{w_{n}}{w_{n}} \end{array}] \end{array}$$

where *w*_1_/*w*_2_ is the relative importance of criterion (or sub-criterion) *i* with respect to criterion (or sub-criterion) *j*. Such relative weights are usually defined according to the well known Saaty’s scale (see Table 7).

**Table 7:** The Saaty’s scale for AHP.

*W*_*ij*_	Definition
1	Equal importance
3	Moderate importance of one over another
5	Essential or strong importance
7	Very strong importance
9	Extreme importance
2, 4, 6, 8	Intermediate values between the two adjacent judgements

In general, *W* is a squared matrix whose dimensions are equivalent to the number of criteria (or sub-criteria) used in the decision-making process.

AHP helps in finding the true values of *w*_*i*_ and *w*_*j*_ based on an estimation of the ratios *w*_*i*_/*w*_*j*_ between each couple of criteria (or sub-criteria), collected in the *n* × *n* comparison matrix. Note that the pairwise matrix depends on the decision maker considered: the elements of the matrix are filled with the individual’s judgment on a comparison between the utility of two criteria (e.g., “how important criterion X is with respect to criterion Y”) expressed as a ratio, rather than directly stating a numerical value for the utility of each decision criterion (i..e, “The importance of X is *α*”). For *local consistency*, if criterion *X* is *x* times more important than criterion *Y*, then it follows that the relative importance of *Y* with respect to *X* is $\frac{1}{x}$. In other words, $W_{ij}=W_{ji}^{-1}$. In these conditions, the decision maker is consistent with respect to individual pairwise comparisons, and all the diagonal elements of *W*_*ii*_ are equal to unity. Briefly, AHP allows to compute the weights *w*_*i*_ of each decision criterion, based on the relative ratios collected in *W*. In particular, the approach proposed by Saaty relies on the fact that, in the ideal case (when the expert judgment *W*_*ij*_ is exactly equal to the ratio *w*_*i*_/*w*_*j*_), the dominant eigenvalue is λ_*max*_(*W*) = *n* and the weight vector $w=[w_{1},\ldots ,w_{n}]$ is the corresponding eigenvector, up to a scaling factor. This happen when relative judgements are *perfectly consistent*, i.e. *W*_*ij*_ = *W*_*ik*_*W*_*kj*_ for each *i*, *j*, *k* = 1, …, *n*. Unfortunately, since real data are typically affected by *inconsistencies*, there is no vector w such that *W*_*ij*_ = *w*_*i*_/*w*_*j*_ for each couple of alternatives, and it is necessary to resort to approximations or compromises solution such as the Logarithmic Least Squares approach [65].

Notice that, standard AHP requires information on all pairs of criteria; this poses a heavy burden on the interviewed subjects, rendering the technique quite impractical when some criteria are hardly comparable or their number is large. To overcome this issue, in the literature, several techniques have been proposed ([66–68]) which are able to handle missing comparisons, i.e., *W* is a sparse matrix with *W*_*ij*_ = 0 when the decision maker is unable to compare some couples of criteria *i*, *j*. In this view, an effective way to represent the sparsely available information is to assume a graph-theoretical perspective, where the criteria play the role of nodes, while the availability of a nonzero entry *W*_*ij*_ corresponds to an edge between criterion *i* and criterion *j*. In order to reconstruct the utilities of the criteria, it is sufficient that the graph *G* obtained as described above is connected (the graph is undirected as we assume that $W_{ji}=W_{ij}^{-1}$).

#### Criteria relevance estimation stage

Among other approaches to solve this problem, one of the most effective ones is the *Incomplete Logarithmic Least-Squares approach* (ILLS) [68], where one aims at finding the vector w* that solves the following optimization problem:

$$\begin{array}{c} w^{*}=\underset{x\in R_{+}^{n}}{\text{arg}\;\text{min}}\{\frac{1}{2}\sum_{u=1}^{g}\sum_{i=1}^{n}\sum_{v_{j}\in N_{i}}{\left(\text{ln}\left(W_{ij}^{\left(u\right)}\right)-\text{ln}(\frac{x_{i}}{x_{j}})\right)}^{2}\}, \end{array}$$ (2)

where $W_{ij}^{\left(u\right)}$ represents the comparison matrix provided by the decision maker *u* and *g* is the number of decision-makers involved in the process. Notice that, the notation $v_{j}\in N_{i}$ is based on the *graph representation* of the sparse comparison matrix. In the graph-theoretical representation, a graph *G* = {*V*, *E*} represents the graph underlying *Y*. In this view, the nodes *V* = *v*_1_, …, *v*_*n*_ correspond to the *n* criteria, while the edges in *E* are associated to the given relative judgments (outside the diagonal), hence (*v*_*i*_, *v*_*j*_)∈*E* ⇔ *Y*_*ij*_ ≠ 0 and *i* ≠ *j*. The notation $v_{j}\in N_{i}$ allows us to consider only the defined ratios in the sparse pairwise comparison matrix. An effective strategy to solve the above problem is to operate the substitution y=ln(x), where ln(⋅) is the component-wise logarithm so that Eq (2) can be rearranged as

$$\begin{array}{c} \begin{array}{cc} w^{*} & =\text{exp}(\underset{y\in R^{n}}{\text{arg}\;\text{min}}\{\frac{1}{2}\sum_{u=1}^{g}\sum_{i=1}^{n}\sum_{v_{j}\in N_{i}}{\left(\text{ln}\left(W_{ij}\right)-y_{i}+y_{j}\right)}^{2}\}), \end{array} \end{array}$$ (3)

where exp(⋅) is the component-wise exponential. Let us define

$$\begin{array}{c} \kappa \left(y\right)=\frac{1}{2}\sum_{u=1}^{g}\sum_{i=1}^{n}\sum_{v_{j}\in N_{i}}{\left(\text{ln}\left(W_{ij}^{\left(u\right)}\right)-y_{i}+y_{j}\right)}^{2}; \end{array}$$

because of the substitution **y** = ln(**x**), the problem becomes convex and its global minimum is in the form w* = exp(y*), where y* satisfies

$$\begin{array}{c} \frac{\partial \kappa (y)}{\partial y_{i}}{|}_{y=y^{*}}=\sum_{u=1}^{g}\sum_{v_{j}\in N_{i}}\left(\text{ln}\left(W_{ij}^{\left(u\right)}\right)-y_{i}^{*}+y_{j}^{*}\right)=0,\;\forall i=1,\ldots ,m. \end{array}$$

Let us consider the *n* × *n* matrix *P*^(*u*)^ such that $P_{ij}^{\left(u\right)}=\text{ln}\left(W_{ij}^{\left(u\right)}\right)$ if $W_{ij}^{\left(u\right)}>0$ and $P_{ij}^{\left(u\right)}=0$, otherwise; we can express the above conditions in a compact form as

$$\begin{array}{c} L\left(A^{\left(u\right)}\right)y^{*}=P^{\left(u\right)}1_{n}, \end{array}$$ (4)

where $L(A^{\left(u\right)})$ is the Laplacian matrix associated to the graph *G*, considering an adjacency matrix *A*^(*u*)^ underlying the comparison matrix according to Eq (5)

$$\begin{array}{c} A_{ij}^{\left(u\right)}=\{\begin{array}{cc} 1 & \text{if}\;W_{ij}^{\left(u\right)}\neq 0\\ 0 & \text{otherwise.} \end{array} \end{array}$$ (5)

Notice that, since for hypothesis *G* is undirected and connected, the Laplacian matrix L(A) has rank *n*−1 [69]. Therefore, we approximate the solution by computing

$$\begin{array}{c} y^{*}=L{\left(A^{\left(u\right)}\right)}^{†}P^{\left(u\right)}1_{n}, \end{array}$$

where $L{\left(A^{\left(u\right)}\right)}^{†}$ is the Moore-Penrose left pseudoinverse of $L(A^{\left(u\right)})$.

Due to the hierarchical structure of the approach based on criteria and sub-criteria, it is necessary to apply the same scheme, based on ILLS, to each level of the hierarchy considering a comparison matrix for each level of the structure. Once obtained the local weights for each criterion and sub-criterion, the global weights are computed as a multiplication between the local weights.

#### Inconsistency evaluation

Unfortunately, comparison matrices are frequently affected by inconsistency (i.e., *W*_*ij*_ ≠ *W*_*ik*_*W*_*kj*_. As Saaty states, due to the lack of accuracy in individuals’ minds, judgments might not be consistent. The *Consistency Index* (*CI*) is the most frequently used metric for the evaluation of the inconsistency degree of a given instance, it is based on the dominant eigenvalue λ_*max*_(*W*) of the comparison matrix $W\in R^{n\times n}$:

$$\begin{array}{c} CI\left(W\right)=\frac{\lambda_{max}\left\{W\right\}-n}{n-1}, \end{array}$$ (6)

where *n* represents the number of considered alternatives. Moreover, Saaty proposed to normalize such index with respect to the so-called *Random Index* (*RI*_*n*_) which is the average *CI*(*W*) computed by considering a large number of random pairwise comparison matrices of degree *n*, thus obtaining the *Consistency Ratio* as in Eq (7).

$$\begin{array}{c} CR\left(W\right)=\frac{CI(W)}{RI_{n}} \end{array}$$ (7)

If *CR* is smaller or equal to 10%, the inconsistency is considered acceptable and the absolute utilities can be computed (e.g. via LLS), if instead *CR* is greater than such a threshold, it is suggested to revise the subjective relative judgment in order to reduce such inconsistency [70]. Notice that such an approach is valid for complete comparison matrices (i.e., when *W*_*ij*_ ≠ 0 for each couple of alternatives or criteria (*i*, *j*)). A similar approach is applied also in the sparse setting (i.e., when *W*_*ij*_ = 0 for some couple of alternatives or criteria (*i*, *j*)). The *Sparse Consistency Index* [71] directly derives from the consistency index proposed by Saaty in Eq (6). In the sparse setting, it is defined as:

$$\begin{array}{c} \tilde{CI}\left(W\right)=\frac{\lambda_{max}\left\{M\right\}-n}{n-1}, \end{array}$$ (8)

where M is an *auxiliary matrix* obtained from the sparse comparison matrix *W* according to one of the procedures described in [67, 71]. Similarly to the complete information setting, the proposed index requires normalization. In fact, in [71] the authors define an alternative Random Index ${\tilde{RI}}_{n,m}$ also for the sparse setting which is a function of matrix size *n* and the number of missing relative comparisons *m*. As a result, the *Consistency Ratio for sparse comparison matrices* $\tilde{CR}$ is defined as:

$$\begin{array}{c} \tilde{CR}\left(W\right)=\frac{\tilde{CI}\left(W\right)}{{\tilde{RI}}_{n,m}}. \end{array}$$ (9)

Without any changes, as in the complete information setting, the rule about the 10% threshold for the ratio $\tilde{CI}/{\tilde{CR}}_{n,m}$ can be adopted.

### Appendix 2—Questionnaire

#### Interviewee profiling

Q1
Genre
  a. Male  b. Female  c. Other
Q2
Age
  a. 18-25  b. 26-35  c. 36-45  d. 46-55  e. 56-65  f. 66-75  g. 76-85  h. 86-95
Q3
Educational qualification
  a. Middle School Certificate  b. High School Diploma  c. Bachelors Degree  d. Master/PhD
Q4
Region of Residence
  a. Specify your Italian region
Q5
Occupation
  a. Stay-at-home partner  b. Trader  c. Manager/Officer  d. Professor/teacher  e. Armed Forces/Guards/Vigilance  f. Employee  g. Entrepreneur  h. Nurse  i. Freelancer—other  l. Freelancer—architect  m. Freelancer—lawyer  n. Freelancer—accountant  o. Freelancer—engineer  p. Physician/dentist/pharmacist  q. Factory worker  r. Retired  s. Researcher  t. Unemployed  u. Student  v. Undefined
Q6
If a vaccine for Covid-19 was available
  a. I would not vaccinate  b. I would vaccinate after a few days  c. I would vaccinate after a few months  d. I would vaccinate right away
Q7
Do you think there is a relationship between Covid-19 and 5G technology
  a. Definitely no  b. Probably no  c. Probably yes  d. Definitely yes  e.I do not know
Q8
How has your job changed?
  a. Same job with lower workload  b. Same job with higher workload  c. I didn’t have a job  d. Same job with same workload  e. I lost the job due to COVID-19
Q9
What sources do you consult to update you about the progress of the epidemic and its effects?
  a. Scientific Articles  b. Social Network Groups  c. Statistical Survey Reports  d. Official bulletins of health care facilities  e. Newswires  f. Newspapers

#### Criteria and sub-criteria comparisons

Q10
Which of these two aspects, health parameters and economic parameters, do you consider most important?
  a. Equally Important  b. Health Parameters  c. Economic Parameters
Q11
How much more important do you consider it to be?
  a. Much more important  b. Definitely more important  c. little more important
Q12
Which of these two aspects, Number of Covid-19 cases and Number of Covid-19 molecular tests, do you consider most important?
  a. Equally Important  b. Number of Covid-19 cases  c. Number of Covid-19 molecular tests
Q13
How much more important do you consider it to be?
  a. Much more important  b. Definitely more important  c. Little more important
Q14
Which of these two aspects, Number of deaths and Number of Covid-19 molecular tests, do you consider most important?
  a. Equally Important  b. Number of deaths  c. Number of Covid-19 molecular tests
Q15
How much more important do you consider it to be?
  a. Much more important  b. Definitely more important  c. Little more important
Q16
Which of these two aspects, Number of ICU beds and Number of Covid-19 molecular tests, do you consider most important?
  a. Equally Important  b. Number of ICU beds  c. Covid-19 molecular tests
Q17
How much more important do you consider it to be?
  a. Much more important  b. Definitely more important  c. Little more important
Q18
Which of these two aspects, Number of deaths and Number of Covid-19 cases, do you consider most important?
  a. Equally Important  b. Number of deaths  c. Number of Covid-19 cases
Q19
How much more important do you consider it to be?
  a. Much more important  b. Definitely more important  c. Little more important
Q20
Which of these two aspects, Number of ICU beds and Number of Covid-19 cases, do you consider most important?
  a. Equally Important  b. Number of ICU beds  c. Number of Covid-19 cases
Q21
How much more important do you consider it to be?
  a. Much more important  b. Definitely more important  c. Little more important
Q22
Which of these two aspects, Number of ICU beds and Number of deaths, do you consider most important?
  a. Equally Important  b. Number of ICU beds  c. Number of deaths
Q23
How much more important do you consider it to be?
  a. Much more important  b. Definitely more important  c. Little more important
Q24
Which of these two aspects, Companies lost revenue and Number of suspected workers, do you consider most important?
  a. Equally Important  b. Companies lost revenue  c. Number of suspected workers
Q25
How much more important do you consider it to be?
  a. Much more important  b. Definitely more important  c. Little more important
Q26
Which of these two aspects, Companies lost revenue and Impact on tourism and transport supply chain, do you consider most important?
  a. Equally Important  b. Companies lost revenue  c. Impact on tourism and transport supply chain
Q27
How much more important do you consider it to be?
  a. Much more important  b. Definitely more important  c. Little more important
Q28
Which of these two aspects, Number of suspected workers and Impact on tourism and transport supply chain, do you consider most important?
  a. Equally Important  b. Number of suspected workers  c. Impact on tourism and transport supply chain
Q29
How much more important do you consider it to be?
  a. Much more important  b. Definitely more important  c. Little more important
